# MCT1-mediated endothelial cell lactate shuttle as a target for promoting axon regeneration after spinal cord injury

**DOI:** 10.7150/thno.96374

**Published:** 2024-09-03

**Authors:** Chaoran Shi, Jiaqi Xu, Yinghe Ding, Xingyi Chen, Feifei Yuan, Fengzhang Zhu, Chunyue Duan, Jianzhong Hu, Hongbin Lu, Tianding Wu, Liyuan Jiang

**Affiliations:** 1 Department of Spine Surgery and Orthopaedics, Xiangya Hospital, Central South University, Changsha, 410008, Hunan Province, China.; 2 Key Laboratory of Organ Injury, Aging and Regenerative Medicine of Hunan Province, Changsha, 410008, Hunan Province, China.; 3 National Clinical Research Center for Geriatric Disorders, Xiangya Hospital, Central South University, Changsha, 410008, Hunan Province, China.; 4 Eye Center of Xiangya Hospital, Central South University, Changsha, 410008, Hunan, China.; 5 Department of Sports Medicine, Xiangya Hospital, Central South University, Changsha, 410008, Hunan Province, China.

**Keywords:** spinal cord injury, endothelial cell, lactate shuttle, neuron metabolism, axon regeneration

## Abstract

**Rationale:** Spinal cord injury (SCI)-induced vascular damage causes ischemia and hypoxia at the injury site, which, in turn, leads to profound metabolic disruptions. The effects of these metabolic alterations on neural tissue remodeling and functional recovery have yet to be elucidated. The current study aimed to investigate the consequences of the SCI-induced hypoxic environment at the epicenter of the injury.

**Methods:** This study employed metabolomics to assess changes in energy metabolism after SCI. The use of a lactate sensor identified lactate shuttle between endothelial cells (ECs) and neurons. Reanalysis of single-cell RNA sequencing data demonstrated reduced MCT1 expression in ECs after SCI. Additionally, an adeno-associated virus (AAV) overexpressing MCT1 was utilized to elucidate its role in endothelial-neuronal interactions, tissue repair, and functional recovery.

**Results:** The findings revealed markedly decreased monocarboxylate transporter 1 (MCT1) expression that facilitates lactate delivery to neurons to support their energy metabolism in ECs post-SCI. This decreased expression of MCT1 disrupts lactate transport to neurons, resulting in a metabolic imbalance that impedes axonal regeneration. Strikingly, our results suggested that administering adeno-associated virus specifically to ECs to restore MCT1 expression enhances axonal regeneration and improves functional recovery in SCI mice. These findings indicate a novel link between lactate shuttling from endothelial cells to neurons following SCI and subsequent neural functional recovery.

**Conclusion:** In summary, the current study highlights a novel metabolic pathway for therapeutic interventions in the treatment of SCI. Additionally, our findings indicate the potential benefits of targeting lactate transport mechanisms in recovery from SCI.

## Introduction

Spinal cord injury (SCI) is the leading cause of premature mortality and enduring disability, posing a significant challenge in contemporary medicine. During 1990-2019, the incidence and prevalence of SCI, as well as the lifespan of individuals with disability, increased significantly 1. The pathophysiology of SCI encompasses a multifaceted process wherein an initial mechanical insult triggers a cascade of secondary damage 2. In addition, the disruption of endothelial cell tight junctions indicates compromised vascular integrity. Such disturbances disrupt the blood-spinal cord barrier (BSCB), augmenting vascular permeability 3, 4.

Subsequently, the injury site continually experiences ischemia and edema 5, which, in turn, exacerbates thrombosis, effectuates vascular spasm, and disrupts homeostasis 6. Thus, metabolic dysfunction is inevitable in cells at the epicenter of injury in this prolonged hypoxic-ischemic pathological environment 7-9. The aberrant alterations in metabolic products after SCI and their impact on nerve regeneration have yet to be elucidated 10. Therefore, managing energy metabolism within the injury epicenter is pivotal for recovery from SCI.

Endothelial cells (ECs) are vital components of the BSCB and regulate selective transport and metabolic exchange between the blood and the spinal cord 11. Although ECs regenerate at the epicenter of injury after SCI, their functional attributes are markedly diminished compared with those of their healthy counterparts, i.e., reduced barrier functionality compromises substance transport 12, 13. Some studies have identified lactate as a metabolic byproduct and an energy substrate and signaling molecule crucial for axon regeneration post-SCI 14, 15. Thus, lactate homeostasis is a critical factor in determining recovery post-SCI. Importantly, lactate shuttles between highly glycolytic cells and adjacent cells in the central nervous system 16. Several studies have confirmed that the astrocyte-neuron lactate shuttle (ANLS) is crucial for memory formation, neuropathic pain, and the exacerbation of neurodegenerative diseases 17-20. Consequently, aerobic glycolysis is increased in astrocytes, which, in turn, triggers their lactate-mediated neuronal activity. However, whether a lactate shuttle exists between spinal cord ECs and neurons and the mechanism underlying lactate-mediated neuronal metabolism post-SCI have yet to be elucidated.

Monocarboxylic transporters (MCTs) are crucial for intracellular and intercellular lactate shuttle transport in the central nervous system 21. A previous study indicated that ECs predominantly express MCT1 in the spinal cord 22. However, whether MCT1 is the primary protein involved in lactate transport in ECs and facilitates intercellular lactate shuttling in the central nervous system has yet to be clarified. Thus, the underlying mechanism and functional implications of the expression of MCT1 in ECs post-SCI need further investigation.

In the present study, metabolomics analysis revealed elevated lactate levels post-SCI. Bioinformatics analysis also revealed that MCT1 expression is reduced in ECs following SCI. Furthermore, *in vitro* coculture assays used a lactate sensor and confirmed the presence of a lactate shuttle between ECs and neurons. Next, we leveraged adenovirus-mediated MCT1 overexpression through *in situ* injection in a mouse model. These findings delineate the pivotal role of MCT1 in modulating energy metabolism, which is crucial for regenerating ECs and axons post-SCI. Together, these findings provide insight into a novel metabolic therapeutic strategy for rehabilitation after SCI.

## Methods

### Cell Lines

Mouse brain ECs (bEnd.3 cells, CL-0598, Procell) were maintained with culture media containing 10% FBS (FBS-CP500, NEWZERUM), 1% Penicillin-Streptomycin Solution (PB180120, Procell) in DMEM (10-013-CVRV, CORNING) at 37 ℃ in a 5% CO_2_-humidified incubator. Cell line was obtained directly from Pricella, with no additional cell authentication performed. Mouse nerve cells (CATH.a cells, HTX2209, ATCC) were maintained in CATH.a cell-specific medium (CM-0665, Procell) at 37 ℃ in a 5 % CO_2_-humidified incubator. Cell line was obtained directly from ATCC, with no additional cell authentication performed.

### Mouse cortical neurons preparation and culture

Before isolating primary cortical neurons, prepare a working solution of poly-L-lysine (P6407, Sigma-Aldrich) at a final concentration of 100 μg/ml using PBS buffer. After coating confocal dishes with poly-L-lysine overnight, rinse them 2-3 times with PBS. Extract the embryonic mice and place the intact brain in pre-chilled Hank's Balanced salt solution (B430KJ, BasalMedia) containing 2% penicillin-streptomycin. Remove the cortical meninges and blood vessels, add trypsin, and incubate in an incubator for 12 min. Add an equal or slightly greater volume of planting medium to stop the digestion and filter the suspension through a 70 μm cell strainer (5021100, Saining). Centrifuge at 1200 rpm for 5 min at 4 °C, discard the supernatant and resuspend the cells in DMEM/F12 complete medium (containing 10% FBS). Gently pipette to mix, count the cells, and adjust the cell concentration to 1×10^5^ cells/ml. The cells were resuspended and cultured with neurobasal medium containing 2% B27, 1% glutamine (Gibco, United States), and 1% penicillin-streptomycin in poly-lysine pre-coated confocal dishes.

### Cell treatment and co-culture

The bEnd.3, CATH.a or cortical neurons were seeded in six-well plates at a density of 1 × 10^5^ cells/ml. We exposed bEnd.3, CATH.a or cortical neurons with 10mM exogenous L-lactate (L6402, sigma), 5 mM α-cyano-4-hydroxycinnamic acid (α-CHCA) (S8612, Selleck), and 5 mM 2-Deoxy-D-glucose (2-DG) (S4701, Selleck) for 1 day before being examined.

For the contact co-culture experiments, Ibidi 2-well inserts (81176, Ibidi) were installed into a 20 mm confocal culture dish (801001, NEST) with sterile tweezers and gently pushed with a gloved fingertip to ensure the insert was firmly seated against the bottom of the dish. Cortical neurons and bEnd.3 cells were cultured at a density of 1 × 10^5^ cells/cm² on opposite sides of the chamber. After the cells adhered to both sides, a complete culture medium containing 10 mM lactate was added only to the bEnd.3 side of the co-culture chamber for 24 hours. After removing the chamber, we replaced the medium with a glucose-free culture medium to ensure that neurons do not take up exogenous lactate. After 48-hour incubation, cells were fixed with freshly prepared 4% PFA (AWI0056b, Abiowell) for 30 min at room temperature. After fixing, cells were subjected to imaging using a Zeiss confocal laser scanning microscope LSM 9.

### Lentiviral vector construction and transfection

The construct for Laconic (Plasmid #44238; San Martin et al, 2013) was obtained from Addgene. Seed well-conditioned target cells at a density of 5 × 10^4^ cells/well. Plate in a 24-well plate, and after cell counting, add 500 µL of DMEM culture medium to each well. After 24 hours, replace with DMEM medium containing 1 µg/mL Polybrene-plus, choosing an MOI (Multiplicity of Infection) value of 40, and add 20 µL of 1 × 10^8^ TU/mL viral particles. After 24 hours, replace with fresh culture medium. 72 hours post viral infection, observe under a fluorescence microscope to evaluate the efficiency of lentiviral infection in the target cells.

### Adeno-associated virus construction and transfection

Adeno-associated virus (AAV) used in this study was produced at the Obio Technology (Shanghai, China). The following TlE1^+^ cell-specific AAV plasmid was used and detailed sequence information is available as detailed or upon request: pAAV-TlE1p-mCherry-P2A-Slc16a1-3xFLAG-WPRE (Obio Technology). The target gene sequence for MCT1/Slc16a1 was designed based on GenBank ID NM_009196.4. All experimental controls received injections of a control AAV virus vector, H28906 pAAV-TIE1p-MCS-mcherry-3xFLAG-WPRE (Obio Technology). Injection volumes, coordinates and experimental purpose are described below.

### Mice

Adult male or female C57BL/6 mice (8-10 weeks old, weight 20-25 g) were used in this study. The animals were purchased from Hunan SJA Laboratory Animal Company Limited and were kept in a pathogen-free animal facility at Central South University, following standard purification procedures. They were housed in the laboratory animal unit, with a 12-hour day/night cycle, and were provided ad libitum access to food and water. All mice are housed in a laboratory animal facility with an ambient temperature of 22-24 °C and relative humidity of 60%-80%. Manual bladder voiding and all other animal care were performed 2 to 4 times daily throughout the entire experiment.

### SCI models

The Animal Care and Use Committee of Central South University, Changsha, China, approved all animal protocols and experimental procedures. Perform routine anesthesia, skin preparation, disinfection, and draping. Make an incision centered on the T10 spinous process, sequentially cutting through the skin, subcutaneous tissue, muscles, and lamina to expose the spinal cord. Severe crush SCI were made after laminectomy of a single vertebra by using No. 5 Dumont forceps (Fine Science Tools, Foster City, CA) without spacers and with a tip width of 0.5 mm to completely compress the entire spinal cord laterally from both sides for 5 seconds. Irrigate the wound, then close the incision in layers, cover it with a dressing, and secure it. Postoperatively, mice are housed individually with standard feed and water available ad libitum. Administer penicillin 20,000 units bi-daily via intramuscular injection for postoperative infection prevention for 3 days. Manually express the bladder 2 to 4 times daily to assist with urination.

### Spinal injections for AAV

To overexpress MCT1 after SCI, partial laminectomy at the T9 spinal level was performed two weeks before SCI, using 30-33G Hamilton needles to bilaterally inject with pAAV-TlE1p-mCherry-P2A-Slc16a1-3xFLAG-WPRE or a control AAV virus vector (0.5 µl per injection) at two depths of 0.8 mm and 0.4 mm below the dorsal surface, spaced 1 mm apart, with each injection maintained for 5 min. Post-injection, the needle was left in the spinal tissue for 15-20 min. During the injection process, care was taken to keep the mice warm. The needle was then slowly withdrawn. The skin was sutured, and the wound was disinfected. For postoperative pain relief, mice were administered carprofen via intramuscular injection daily for 3 days. Mice were perfused 28 days after the AAV injection.

### Perfusions

At different time points post-SCI, 1% sodium pentobarbital (50 mg/kg) is administered intraperitoneally for deep anesthesia in mice. Rapid thoracotomy is performed to expose the heart, and a size 12 medical intravenous catheter is inserted into the left ventricle up to the origin of the ascending aorta and secured. The right atrium is incised, followed by sequential perfusion with pre-warmed heparinized saline and 10% formalin solution, maintaining the 10% formalin perfusion for approximately one hour. The specimens are then stored in a 4 °C refrigerator.

### Immunofluorescence

For immunofluorescence of tissue, frozen sections of 16-μm thickness from the spinal cord containing the lesion site were harvested at different time points post-SCI (or sham group) and cut along the sagittal plane. The frozen sections were rewarmed at room temperature for 15 min and rinsed three times with a PBS solution for 10 min each time. The immunohistochemical pen was used to circle the spinal cord sections on the slides. The sections were then permeabilized with 100 μL of PBS solution containing 0.1% Tween 20 and 0.3% Triton-X 100 for 30 min and blocked with 5% BSA in PBS for an additional 30 min. After blocking, the sections were incubated with a group of primary antibodies including F4/80 (Abcam, ab6640, 1:400), TUJ1-1 (CST, 5568, 1:400), Neurofilament-L (CST, 2837S, 1:400), GAP43 (CST, 8945S, 1:400), GFAP (Invitrogen, 130300, 1:500), MCT1 (Proteintech, 20139-1-AP, 1:200), CD31(R&D, fab3628G, 1:200), IBA1 (Wako, 011-27991, 1:500) overnight at 4℃. After being rinsed five times for 10 min each with PBS containing 0.1% Tween 20, the sections were incubated for one hour at room temperature with species-appropriate secondary antibodies conjugated with Alexa Fluor 594 (Abcam, 1:400) or Alexa Fluor 488 (Abcam, 1:400). Subsequently, the sections were rinsed with PBS containing 0.1% Tween 20 before being mounted on slides and covered with DAPI (Genetex).

For immunofluorescence of cells, we place sterile 24-well slides into a 24-well plate. The bEnd.3 or CATH.a, post trypsinization, are seeded onto the slides at a density of 5 × 10^4^ cells/well, with 0.5 ml of complete culture medium added per well. The culture plate is then incubated in a 37 °C, 5% CO_2_ incubator. Once cells reach 85% confluency, the medium is aspirated, and the old culture medium is washed off with PBS along the walls of the well. Each well is then fixed with 500 µl of 4% paraformaldehyde for 12 min, followed by a PBS wash to remove the paraformaldehyde. Each well is treated with 200 µl of 0.1% Triton-X100 in PBST for 20 min. After aspiration of the PBST, the wells are blocked with 3% BSA in PBS solution for 30 min. The blocking solution is then aspirated, and primary antibodies including CD31, MCT1, and TUJ1 are added and incubated overnight at 4 °C. The next day, primary antibodies are aspirated, and the wells are washed three times with PBS for 5 min each. Corresponding secondary antibodies are then added and incubated at room temperature for 1.5 hours. After aspiration of the secondary antibodies, the wells are washed three times with PBS for 5 min each. A drop of DAPI solution containing an anti-fade agent is then added to the slide, and the cell slide is picked up with forceps and inverted onto the slide. The sections were analyzed under a fluorescence microscope or confocal microscope (Zeiss). To validate antibody specificity and distinguish genuine target staining from the background, secondary antibody-only controls were employed. The ImageJ software was used for quantitative analysis of the images, while the Imaris 9.0 software was used for 3D reconstruction.

### Cell lateral migration assay

Firstly, use a Mark pen to draw lines on the backside of a six-well plate to determine the scratch positions. Seed bEnd.3 cells in the six-well plate, with approximately 200,000 cells per well. Allow the cells to grow and fully cover the surface of the six-well plate, then replace the medium with a serum-free culture medium for 6 hours to induce starvation. Use a 200 μl pipette tip, held perpendicular to the cell surface, to draw lines according to the positions marked by the marker pen. Subsequently, wash away the detached cells with PBS, followed by replacing the culture medium with fresh serum-free medium. Incubate the cells in a constant-temperature CO_2_ incubator at 37 °C for 12 hours. Use an optical microscope to observe cell migration at 0 and 24 hours at the scratch site and calculate the migration distance using Image J software.

### Tube formation assay

When bEnd.3 cells reach approximately 85% confluence, the serum-containing medium is replaced with serum-free medium to induce cell starvation for 5 hours. Subsequently, after removing the medium and digesting with trypsin, cells are centrifuged at 1000 rpm for 5 min. The experimental and control groups of bEnd.3 cells are adjusted to a concentration of 80,000 cells/ml using serum-containing culture medium based on cell counting. Pre-cooled pipette tips are used to evenly coat a 96-well plate with Matrigel matrix gel at 100 μl per well, and the plate is then incubated in a constant-temperature incubator for 30 min. Once the matrix gel solidifies, 500 μl of cell suspension is added to each well of a 24-well plate and incubated in a constant-temperature incubator for 6 hours. The formation of capillary-like structures by the cells is observed using an optical microscope, and Image J software is employed for quantitative analysis of the tube formation.

### *In vitro* neurite outgrowth assay

Following the previously described method, primary cortical neurons were extracted and seeded in a 10 mM lactate and 5 mM 2-DG environment to investigate their ability to promote neurite outgrowth. At least six random fields were measured for each group, with all measurements conducted by personnel blinded to the treatment conditions. Neurite outgrowth in cortical neurons under different treatment conditions was assessed using β-III-tubulin (TUJ1) immunofluorescence staining, capturing fluorescent red images (showing cell bodies and neurites) for analysis. Quantification of TUJ11-positive cells in stained neurons was performed using ImageJ software and an immunofluorescence microscope (Zeiss, Germany).

### The axonal regenerative assay

Following the previously described method, primary cortical neurons were extracted and seeded onto a microfluidic chip with compartmentalized neuronal culture using silicon-based microfluidics (SND450, Xona Microfluidics) to investigate their ability to promote neurite outgrowth. Primary cortical neurons were firstly seeded into the left culture chamber of the microfluidic chip. When their axons were observed passing through the middle channel, a pipette connected to a negative pressure suction device was placed at the right culture chamber for 5-10 seconds, near the right side of the middle channel. Axons are severed when the fluid bubbles pass through. Aspirate until all media from the opposite reservoir are removed and the right culture chamber is emptied. Quickly add 150 µL of complete medium containing lactate or α-CHCA to the empty chamber. Subsequently, ECs were added exclusively to the left culture chamber or in combination with α-CHCA, after three days of culturing, axon regrowth in the right culture chamber was assessed through β-III-tubulin (TUJ1) immunofluorescence staining. For detailed experimental procedures, refer to the previous method 23.

### Time-lapse imaging

Add complete medium containing 10 mM lactate to the confocal dishes with cortical neurons- bEnd.3 co-culture 30 min before imaging. Perform time-lapse imaging once the bEnd.3 cells and neurons are in contact. Use the Zeiss Celldiscoverer7 LSM900 live-cell confocal microscope in Time series mode, setting the duration to 30 min with intervals of 30 seconds between cycles for live-cell real-time imaging. Export the video from the ZEN software after the experiment.

### Corticospinal tract tracing

The procedure of tracing the corticospinal tract (CST) follows the protocol described in this published paper 24. A vertical midline incision was made from between the eyes to the posterior skull. Using the bregma as the reference point in both the x and y planes, bilateral windows (5 mm in length and 2 mm in width) were created with the medial edges of the windows 0.5 mm lateral to the bregma. Using a digital stereotactic injector (Item: 51709, Stoelting Co. USA), 0.5 µl of biotin-dextran amine (BDA; MW 10,000; 10% in PBS; Molecular Probes) was injected into one of the 10 total sites (5 sites per side). Mediolateral coordinates: 1.5 mm lateral to the bregma; anteroposterior coordinates from the bregma: -1.0, -0.5, 0, -0.75, and -1.5 mm; dorsoventral coordinates: 0.5 mm from the cortical surface; injection rate: 0.1 µl/minute. After each injection was completed, the injector tip was left in place for an additional 5 min to ensure adequate tissue penetration of the BDA solution. After 28 dpi, mice were anesthetized and perfused with 4% paraformaldehyde to detect CST distribution in the spinal cord.

### Western blotting

We utilize a BCA Protein Assay Kit for protein concentration normalization. After adjusting protein samples to a loading buffer with a ratio of 4:1, we denature the proteins in a 95 °C metal bath for 10 min, followed by storage at -20 °C for future use. During electrophoresis, samples are loaded into the gel wells. The voltage is set to 90 V for the first 30 min, then adjusted to 120 V until the protein bands are fully separated. Subsequently, PVDF membranes are activated with anhydrous ethanol and carefully placed on the SDS-PAGE gel, ensuring no bubbles are formed. The PVDF membrane and SDS gel are sandwiched between sponges and filter paper and electrotransferred in an ice-water mixture at 300 mA for 90 min. The PVDF membrane is then blocked with 5% non-fat milk in TBST solution on a shaker for 1.5 hours. Afterward, the membrane is washed three times with TBST, each for 5 min. The corresponding bands are cut, and primary antibodies are added for incubation at 4 °C overnight. The next day, the primary antibodies are aspirated, and the membrane is washed three times with TBST for 10 min each. Corresponding Western blot secondary antibodies are then added and incubated at room temperature on a shaker for 1 hour. After aspiration of the secondary antibodies, the membrane is washed three times with TBST for 10 min each. Finally, using the chemiluminescence reagent (ShareBio, SB-WB001), the immunoreactive bands were visualized with a ChemiDoc XRS Plus luminescent image analyzer (Bio Rad, England). The image analysis was performed using ImageJ software, and the relative expression levels of the target proteins to β-actin were used for statistical comparison.

### Electrophysiology

Capture the mice to be tested and anesthetize with 0.3% sodium pentobarbital. Shave, disinfect the animal, and securely fixate it in a stereotaxic apparatus, maintaining body temperature with a heating pad. Perform a craniotomy to expose the M1 area of the motor cortex. Insert stimulation electrodes, guided by the stereotaxic device, to a depth of 700-1000 mm from the brain surface, targeting corticospinal neurons in the sensorimotor cortex. Position recording electrodes to penetrate the contralateral thigh's sciatic nerve distal end to record muscle action potentials induced by electrical stimulation. Amplify and record bioelectrical signals using the BL-420F Biological Function Experiment System. Stimulation parameters are as follows: stimulation type: micro-voltage, mode: single pulse; delay 100 ms; frequency 100 Hz, stimulation intensity: 14 V.

### BMS score evaluation, LSS swimming test

The recovery of hindlimb motor function in SCI mice is evaluated using the Basso Mouse Scale (BMS) scoring system 24. Assessments are conducted pre-injury and on days 1, 3, 7, 14, and 28 dpi. The BMS score ranges from 0 to 9, with 0 indicating complete paralysis and 9 indicating normal hindlimb motor function. Before each scoring session, mice are placed on the testing platform in advance to acclimate to the environment. Each mouse is observed for a duration of 4 min.

Mice underwent three consecutive days of swimming training in a 5×15 cm water tank, moving from one side to the other. Surgery was performed after the completion of training. On 28 dpi, mice were placed in the same water tank and observed for 30 seconds. The Louisville Swim Scale (LSS) was used to evaluate the swimming abilities of the mice, including assessments of hindlimb movement, hindlimb alternation, forelimb reliance, trunk stability, and body angle 25.

All experiments were conducted with the mice being randomly assigned by two trained observers blinded to the group allocations. Both observers simultaneously observed and recorded the mice's BMS scores, and LSS scores. The final score was determined as the average of the scores given by the two observers.

### Bioinformatic analysis of the single-cell transcriptomic dataset

The single-cell RNA sequencing data of the contusive mouse SCI (GSE162610) and the information on the corresponding annotation were retrieved from the GEO database 25. Data processing and analysis were performed using the R package "Seurat" 26. The genes expressed in less than 10 cells were excluded. The gene expression matrix was normalized and scaled. We selected the top 20 principal components by performing PCA based on 3,000 variable genes. The FindNeighbors and FindClusters functions were used to cluster cells on a shared-nearest-neighbor graph. We visualized the expression level of interested genes using violin plots. The single-nucleus RNA sequencing data of the human adult spinal cord from seven donors (GSE190442) were also obtained and underwent consistent analysis workflow 27. We used the Kruskal-Wallis non-parametric test to compare the expression levels of Slc16a1 at different time points to determine if there were statistically significant differences. Visualization was performed using violin plots.

### Statistical analysis

The statistical analysis of the results was performed using GraphPad Prism (version 7.0, USA). All data were reported as mean ±standard deviation (SD). Normality was determined using the Shapiro-Wilk test. For group number = 2, the homogeneity of variances was tested using the F-test. When the data followed a normal distribution and homogeneity of variance, an unpaired t-test was conducted for the statistical analysis. For group number > 2, the homogeneity of variances was tested using the Brown-Forsythe test. When the data followed a normal distribution and homogeneity of variance, an ordinary one-way ANOVA and Tukey's multiple comparisons test were performed for the statistical analysis. Two-way ANOVA was utilized for data with two variables, grouping and time.

All differences among and between groups were considered statistically significant at p < 0.05. In the figures, ns denotes p ≥ 0.05, ∗ denotes p < 0.05, ∗∗ denotes p < 0.01, ∗∗∗ denotes p < 0.001, and ∗∗∗∗ denotes p < 0.0001.

## Results

### Enhanced glycolytic metabolism after SCI

**Figure 1:**
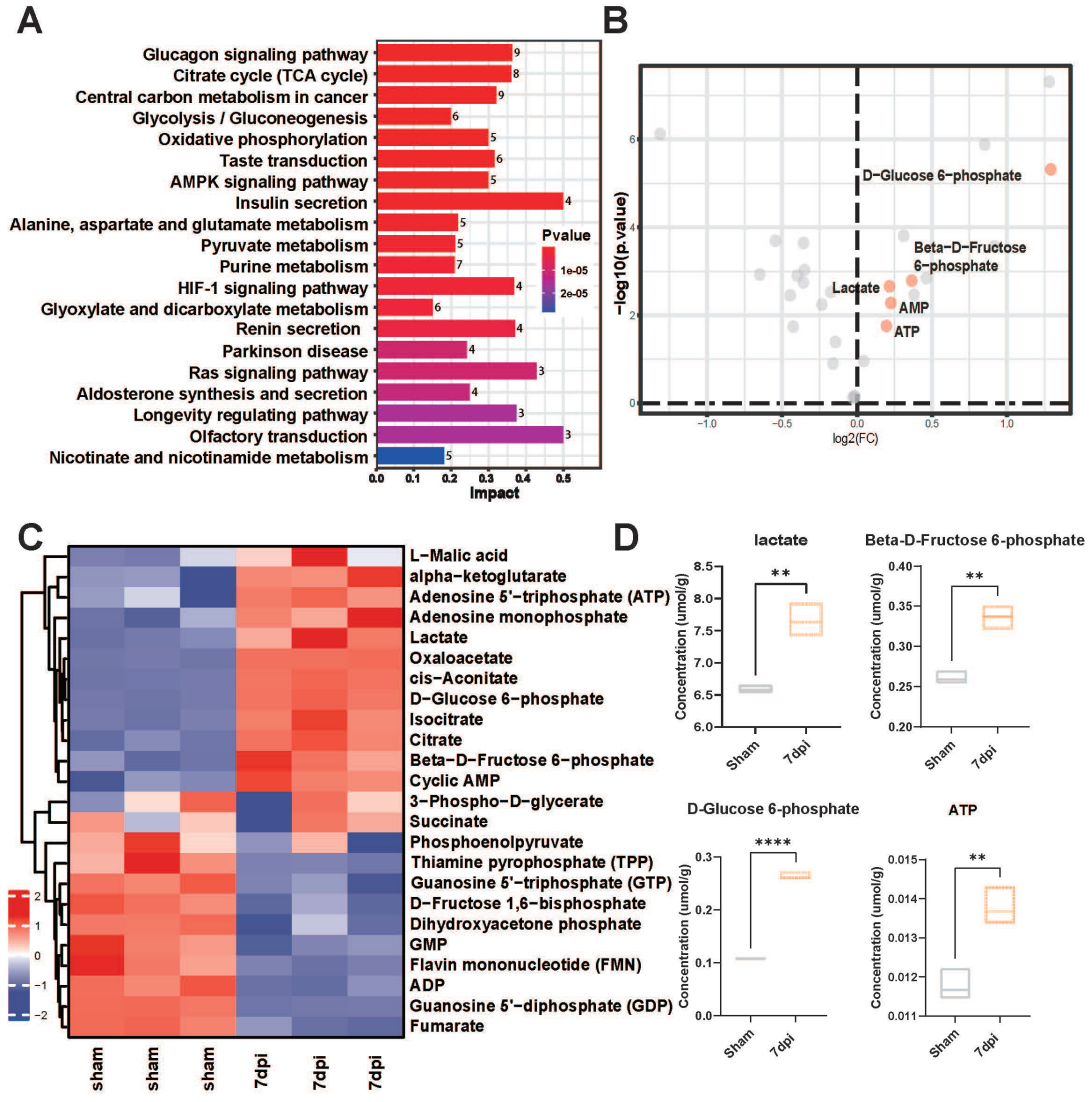
**Precision-targeted metabolomics analysis identified the most affected functional metabolites after SCI. (A)** KEGG analysis of 32 metabolites with heatmap to examine the metabolic pathways with significant changes in energy metabolism after SCI. **(B)** Comparative analysis of 32 energy metabolism metabolites in spinal cord tissues with volcano plotting to identify five glycolysis-related functional metabolites (red points) that were mostly changed after SCI (|log2FoldChange| > 0.1 and P-value < 0.01). **(C)** Heatmap overview of the most significantly differential metabolites in the epicenter of injury tissue that were clearly changed after SCI. **(D)** Violin plot shown the expressional levels of four glycolysis metabolism-related metabolites that were mostly alternated after SCI. (n = 3 for each group, **p < 0.01, ****p < 0.0001).

Accumulating evidence indicates a crucial role of glycolysis in the survival and regeneration of neurons in *Drosophila* 14, 28; however, its role in SCI mice has yet to be investigated. We also collected *in situ* spinal cord tissue samples from mice 7 days after SCI. Targeted metabolomics sequencing was carried out to compare the changes in glycolysis-related metabolites before and after SCI. The heatmap revealed significantly increased metabolites, such as lactate, D-glucose-6-phosphate, b-D-fructose-6-phosphate, and adenosine monophosphate, after injury (**Figure 1A-B**). Additionally, the Kyoto Encyclopedia of Genes and Genomes (KEGG) analysis revealed that glycolysis pathway-related metabolites were upregulated in response to SCI (**Figure 1C**). Concurrently, volcano plots revealed a marked increase in the levels of glycolysis-related metabolites, such as lactate, D-glucose-6-phosphate, and β-D-fructose-6-phosphate, post-injury (**Figure 1D**). Therefore, lactate, a core molecule of glycolysis, was deemed a critical metabolite post-SCI in subsequent experiments.

### Endothelium-derived lactate feeds nerve cells

**Figure 2:**
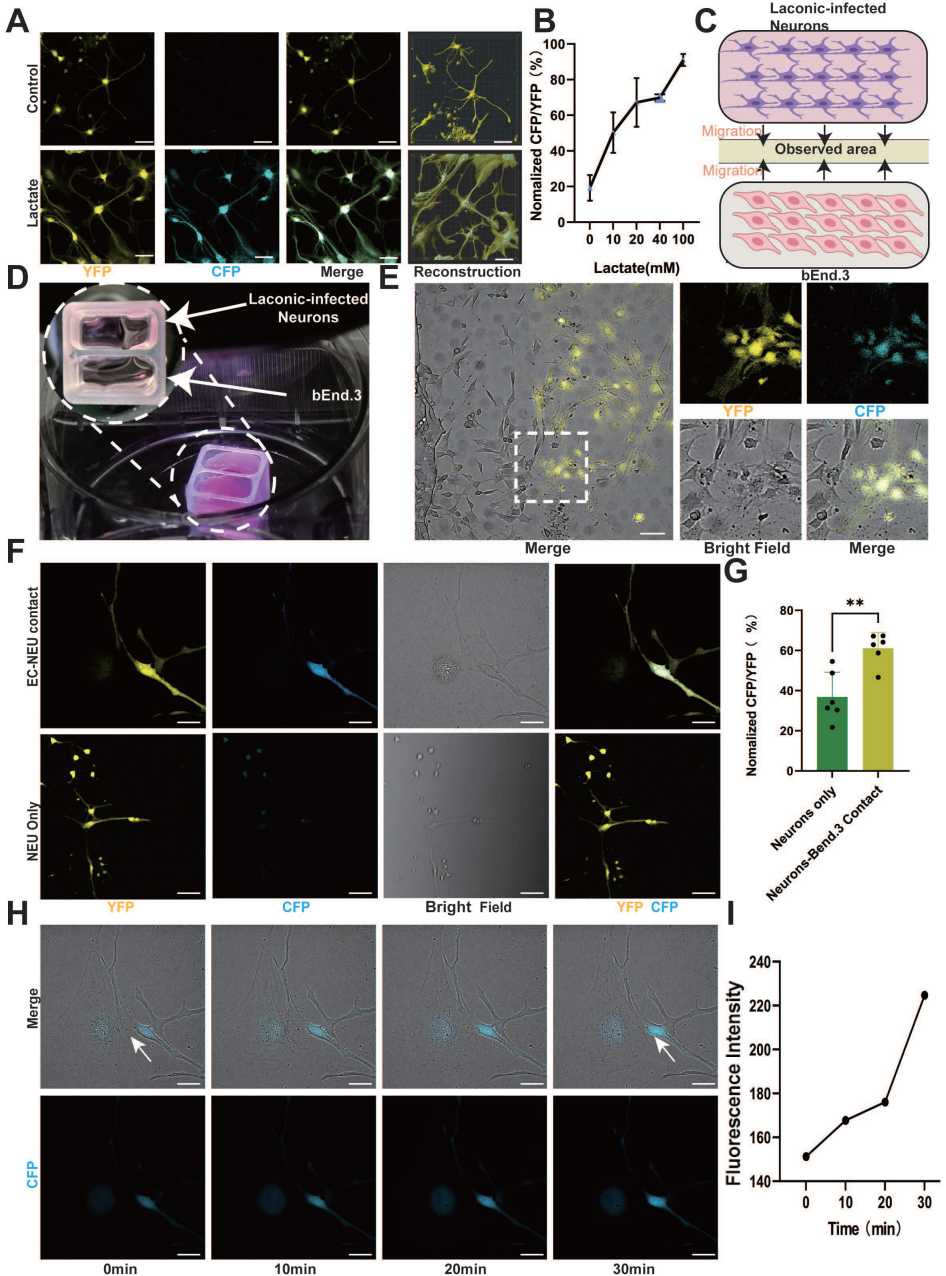
**Endothelium-derived lactate feeds nerve cells. (A)** Representative images showing CFP (blue) and YFP (yellow) fluorescence in control and lactate (100 mM)-treated laconic infected cortical neurons (scale bar = 10 μm).** (B)** Quantification of the CFP/YFP fluorescence ratio of laconic (lactate sensor) showing concentration-dependent lactate uptake in cortical neurons (n = 6 from 3 independent experiments. Error bars indicate the standard error of the mean (SEM)). **(C)** Schematic illustration for the lactate uptake assay after chamber removal. At 24 h after chamber removal, the fluorescence of CFP and YFP were observed in the middle of the wells (yellow area).** (D)** Representative images for the lactate uptake assay with 2-well chamber. Cells were seeded on a glass bottom dish. bEnd.3 and adenoviral laconic-infected cortical neurons were separated by 2-well chamber. After attachment, the 2-well chamber was removed for cell migration. **(E)** Representative confocal image showing CFP (blue) and YFP (yellow), Bright field (white) fluorescence from bEnd.3 and laconic-infected cortical neurons on a 2-well chamber (scale bar = 100 μm). **(F)** Localized enlargements showing CFP (blue) and YFP (yellow), Bright field (white) fluorescence from bEnd.3 and laconic-infected cortical neurons on a 2-well chamber. The upper panel shows cortical neurons with bEnd.3 contact and the lower panel shows cortical neurons without bEnd.3 contact (scale bar = 20 μm). **(G)** Quantification of the CFP/YFP fluorescence ratio of laconic in cortical neurons with bEnd.3 contact (EC-NEU contact) or cortical neurons alone (NEU only) (n = 6 from 3 independent experiments. Error bars indicate the standard error of the mean (SEM) from unpaired Student's t-test, ∗∗ p < 0.01). **(H)** Representative time-lapse imaging at 0, 10, 20, and 30 min, along with CFP (blue), Bright field (white) fluorescence from bEnd.3 and laconic-infected cortical neurons on a 2-well chamber (scale bar = 20 μm). **(I)** Quantification of the CFP fluorescence ratio of laconic showing time-dependent lactate transport from bEnd.3 to cortical neurons.

ECs regulate metabolic exchange between the blood and spinal cord parenchyma, forming a crucial component of the neurovascular unit alongside neurons 11, 29. Moreover, ECs can produce and release a significant amount of lactate and are proximal to neurons; hence, we investigated the role of EC lactate in neuron biology. To examine whether neurons utilize EC-derived lactate, we employed Laconic, a Förster Resonance Energy Transfer (FRET)-based quantitative intracellular lactate sensor 30. Mouse cortical neurons were infected with a lentivirus expressing Laconic and cultured in the presence of a glycolysis inhibitor (2-deoxy-D-glucose, 8 mM) to reduce the production of basal glycolytic lactate. Supposedly, the increase in lactate concentration from 10 mM to 100 mM led to a concentration-dependent increase in the yellow fluorescence protein/cyan fluorescence protein (CFP/YFP) fluorescence ratio (**Figure 2A-B and Figure S1A**). Additionally, we utilized Laconic lentivirus to infect CATH.a. The results also revealed an increase in the CFP/YFP fluorescence ratio in a lactate concentration-dependent manner (**Figure S1B-C**). Next, we cocultured bEnd.3 cells and cortical neurons in a two-well cell culture dish to investigate the neuronal uptake of EC-derived lactate (**Figure 2C**). BEnd.3 and laconic-infected cortical neurons were seeded in separate wells of the culture dish for 24 h for cell adherence, after which the culture insert was removed to allow cell migration (**Figure 2D**). After an additional 24 h, we observed a significant increase in the CFP/YFP fluorescence ratio in cortical neurons that were in direct contact with bEnd.3 cells (**Figure 2E-G**). Similarly, coculture of bEnd.3 cells with CATH.a cells resulted in an increased CFP/YFP fluorescence ratio in CATH.a cells that were in direct contact with bEnd.3 cells, whereas the fluorescence ratio remained at baseline in cortical neurons not in contact with CATH.a cells (**Figure S1D-F**). In order to investigate whether ECs transfer lactate to neurons through direct contact, we used time-lapse imaging and observed real-time fluorescence changes in Laconic lentivirus-infected neurons cocultured with bEnd.3 cells. The imaging results revealed a gradual increase in the intensity of CFP fluorescence in cortical neurons that adhered to bEnd.3 cells, whereas no significant change was detected in cortical neurons that were not in contact with bEnd.3 cells (**Figure 2H-I, Figure S1G-H and Movies S1-2**). These findings indicate that direct contact facilitates lactate transfer from ECs to neurons. Thus, we can deduce that direct contact between ECs and neurons facilitates lactate entry into neurons.

### Deficiency of MCT1 in ECs after SCI

**Figure 3:**
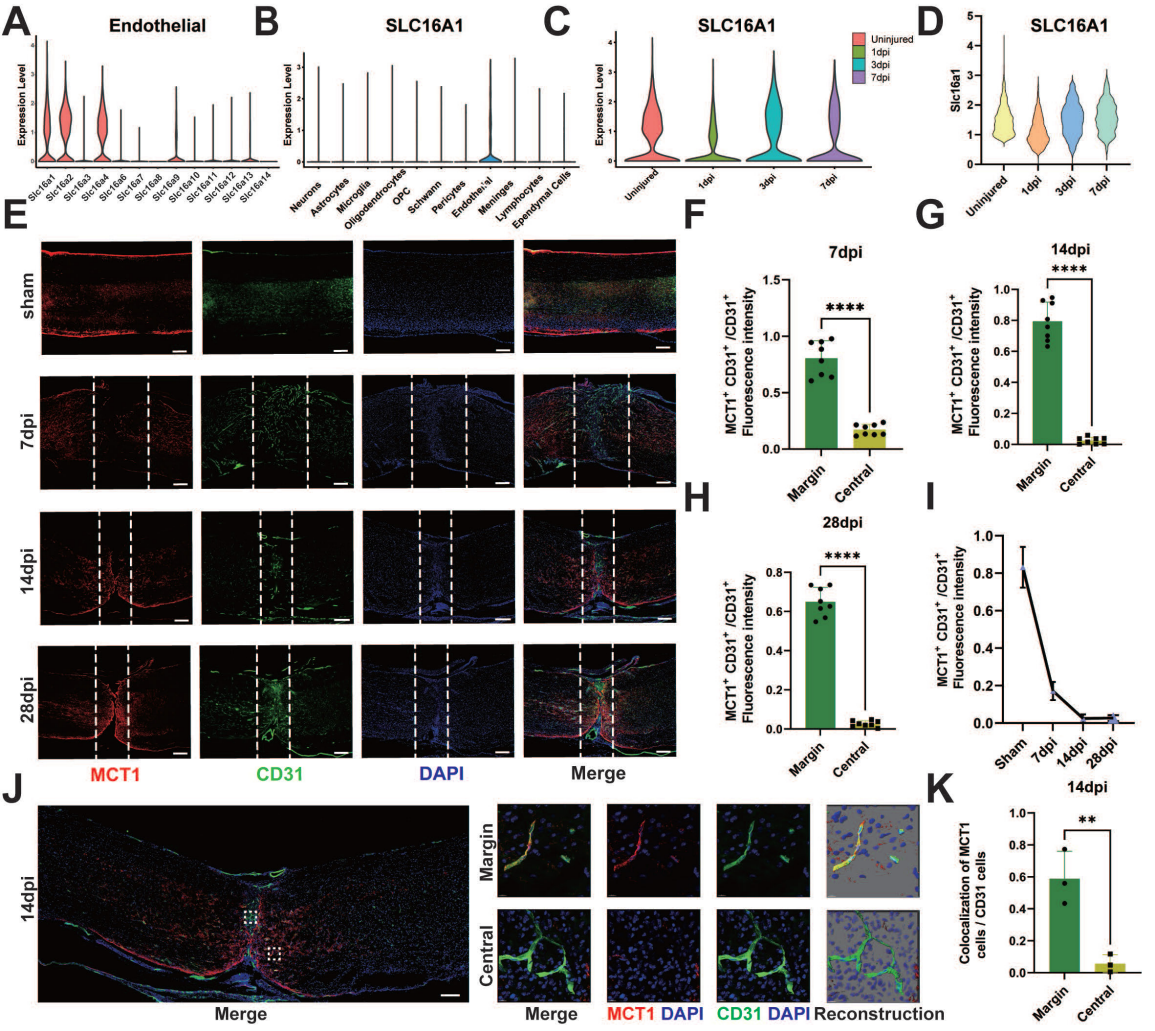
**Spatial and temporal expression of MCT1 after SCI. (A)** The expression level of MCTs in endothelial cells, in single-cell dataset GSE190442. **(B)** The expression level of SLC16a1 (MCT1) in different cell types, in single-cell dataset GSE162610. **(C)** The expression level of SLC16a1 in different time point post-injury, in single-cell dataset GSE162610.** (D)** Violin plot shows the expression level of Slc16a1 in ECs cluster at uninjured mice, 1 dpi, 3 dpi, 7 dpi. **(E)** Representative fluorescence images showing the expression of MCT1 in the spinal cord of the sham and 7, 14, 28 days post-injury (dpi) groups (red: MCT1, green: CD31, blue: DAPI, scale bar: 200 μm). **(F-H)** Quantification of the MCT1^+^ CD31^+^ / CD31^+^ fluorescence intensity in the central and margin regions of 7, 14, and 28 dpi groups in (D) (n = 8 from 6 independent experiments, mean ± SD, unpaired t-test, ****p < 0.0001). **(I)** Quantification of the MCT1^+^ CD31^+^ / CD31^+^ fluorescence intensity in the central regions of 7, 14, and 28 dpi groups in (D) (n = 8 from 6 independent experiments. Error bars indicate the standard error of the mean (SEM)). **(J)** Representative fluorescence images showing the colocalization of CD31 with MCT1 in the spinal cord at 14 dpi (scale bar: 200 μm). Localized enlargements of fluorescence confocal images. The upper panel shows the margin region of the injury site, and the lower panel shows the central region of the injury site (red: MCT1, green: CD31, blue: DAPI, scale bar: 10 μm). **(K)** Quantification of the Colocalization of MCT1 cells with CD31 cells in the central and margin regions in (I) (n = 3 from 3 independent experiments, mean ± SD, unpaired t-test, **p < 0.01).

The elevated levels of lactate in the epicenter of SCI may be associated with deficient lactate transport mechanisms in damaged ECs. Therefore, we investigated the altered transport proteins in spinal cord vascular ECs post-SCI. Lactate transport is mediated primarily by MCTs, also known as the Slc16a family 31. Analysis of existing databases encompassing the adult human spinal cord 27 revealed high expression of MCT1, 4, 5, and 7 (SLC16a1, SLC16a2, SLC16a4) in adult ECs (**Figure 3A**). Interestingly, MCT1 was predominantly expressed in ECs in the spinal cord (**Figure 3B**), prompting an investigation into the role of this protein in endothelial-to-neuronal lactate shuttling. The analysis of single-cell data from SCI mice 25 revealed significantly reduced expression of MCT1 post-SCI (**Figure 3C-D**). Additionally, the current literature indicates a crucial role of MCT1 in the transmembrane transport of lactate, pyruvate, and ketone bodies 32, which is crucial for lactate homeostasis in the spinal cord. Furthermore, the spatiotemporal changes in MCT1 post-SCI were assessed via immunofluorescence. The results revealed the colocalization of MCT1 and CD31 in the spinal cord specimens of the sham-operated mice (**Figure 3E**). However, on 7 days post-injury (dpi), the newly formed blood vessels at the epicenter of injury did not express MCT1, suggesting a potential partial loss of function in these blood vessels (**Figure 3F**). Similarly, immunofluorescence did not reveal MCT1 expression on days 14 and 28 dpi in the newly formed blood vessels (**Figure 3G-H**), but elevated MCT1 expression was noted in the areas surrounding the injury epicenter, albeit without extensive co-staining with CD31 (**Figure 3E, 3I**). To clarify the specific reason for this increase, immunofluorescence revealed some co-staining of MCT1 with GFAP, which was consistent with previous findings 18, 33, 34 (**Figure S2A-D**). Confocal microscopy confirmed the lack of MCT1 expression in ECs at the epicenter of SCI in mice (**Figure 3J-K**). Together, these findings indicate that the newly formed ECs post-SCI exhibit functional loss and decreased expression of MCT1.

### Deficiency of MCT1 in ECs inhibits angiogenesis and glycolysis

**Figure 4:**
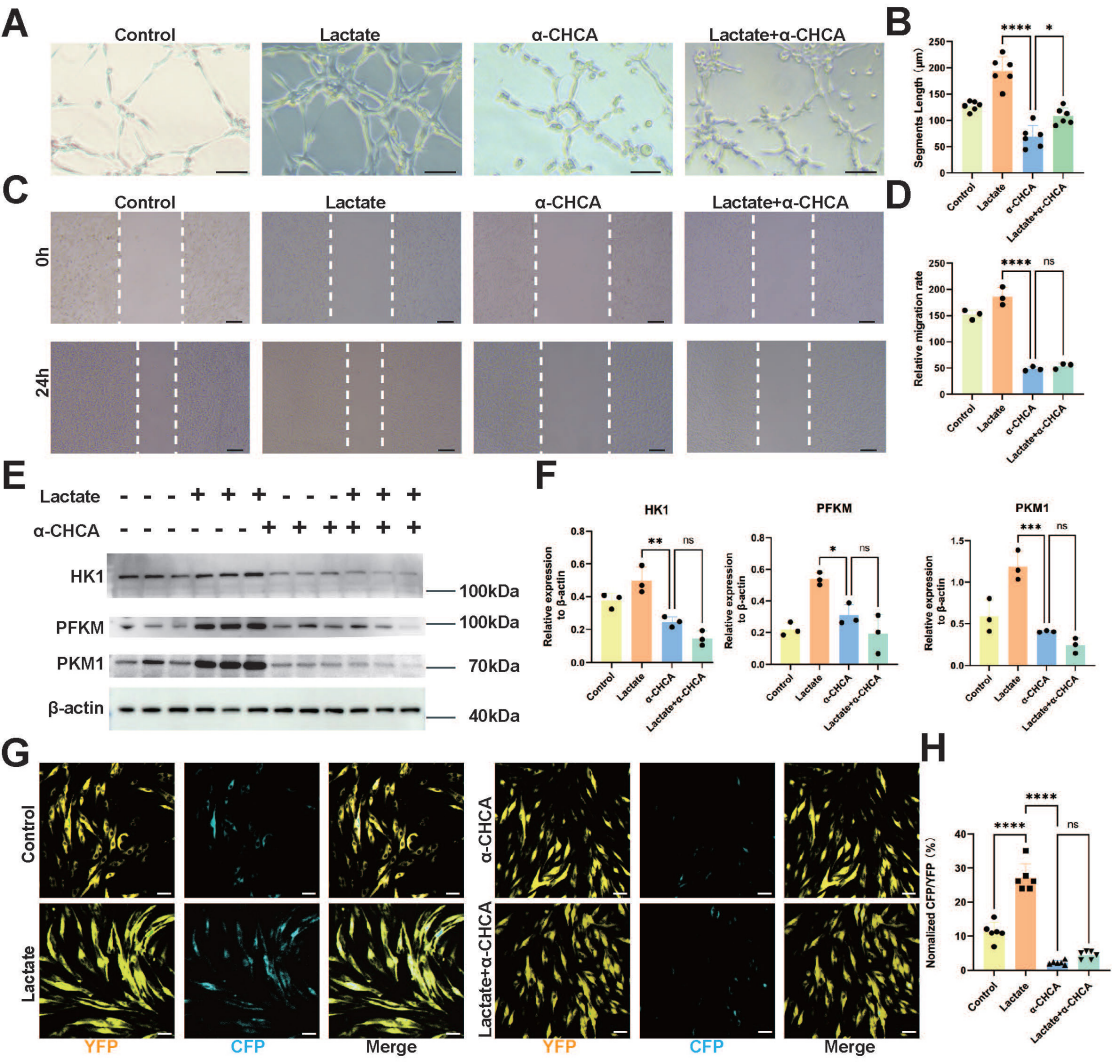
** Inhibition of ECs MCT1 Expression Leads to Decreased Biological and Metabolic Functions. (A)** Representative images showing the tube-forming ability of bEnd.3 Cells under lactate or α-CHCA treatment in four groups (scale bar = 100 μm). **(B)** Quantification of tube-forming ability in (A). (n = 6 from 3 independent experiments, mean ± SD, one-way ANOVA, *p < 0.05, ****p < 0.0001).** (C)** Representative images showing the lateral migration ability of bEnd.3 Cells under lactate or α-CHCA treatment in four groups (scale bar = 100 μm). **(D)** Quantification of tube-forming ability in (C) (n = 3 from 3 independent experiments, mean ± SD, one-way ANOVA, ns not significant, ****p < 0.0001). **(E)** Western blotting analysis of the levels of HK1, PFKM, PKM1 to β-actin in bEnd.3 Cells with different treatments. **(F)** Quantification of the relative expression of HK1, PFKM, PKM1, and β-actin in (E) (n = 3 from 3 independent experiments, mean ± SD, one-way ANOVA, ns not significant, *p < 0.05, **p < 0.01, ***p < 0.001). **(G)** Representative images showing CFP (blue) and YFP (yellow) fluorescence laconic-infected bEnd.3 Cells under lactate or α-CHCA treatment in four groups (scale bar = 10 μm).** (H)** Quantification of the CFP/YFP fluorescence ratio in (G) (n = 6 from 3 independent experiments, mean ± SD, one-way ANOVA, ns not significant, ****p < 0.0001).

To simulate the metabolic environment associated with *in vivo* SCI and elucidate the relevant functions of ECs *in vitro*, we conducted tube formation and transverse migration experiments (scratch assays) in addition to investigating the impact of MCT1 on the biological functions of ECs. Compared with those in the control group, no significant changes in the number or branching length of EC tubes were detected after lactate treatment. However, Western blot assays revealed significant downregulation of MCT1 expression after treatment with the MCT1-specific inhibitor alpha-cyano-4-hydroxycinnamic acid (α-CHCA) (**Figure S3A-B**), as well as a decrease in both the number of cell tubes and their branch length. Lactate addition did not significantly improve tube formation in ECs after MCT1 downregulation (**Figure 4A-B**). Additionally, the lateral migration of lactate-treated ECs did not differ significantly from that of control ECs, whereas the inhibition of MCT1 expression significantly reduced the lateral migration distance of ECs (**Figure 4C-D**). Concurrently, the metabolic status of bEnd.3 cells cultured in isolation were assessed on the basis of the effect of lactate treatment on the expression of key enzymes related to glycolysis, hexokinase 1 (HK1), phosphofructokinase 1 (PFKM), and pyruvate kinase 1 (PKM1), after lactate treatment. Western blotting revealed increased expression of glycolytic enzymes in ECs after lactate treatment. However, the addition of α-CHCA resulted in a decrease in the expression of glycolytic enzymes in ECs, and the simultaneous addition of lactate and α-CHCA did not improve the inhibition of glycolysis (**Figure 4E-F**).

Next, we determined the specific role of MCT1 as a lactate transporter in ECs via the use of Laconic lentivirus-transduced bENd.3 cells. Virus transduction was confirmed by immunofluorescence, and the CFP/YFP fluorescence ratio increased in a lactate concentration-dependent manner (**Figure S4A, D**). Lactate, α-CHCA, and a combination of both treatments were subsequently administered. Immunofluorescence revealed that lactate treatment significantly increased the CFP/YFP fluorescence ratio in bEnd.3 cells, whereas α-CHCA treatment resulted in a marked decrease in the CFP/YFP fluorescence ratio. Moreover, simultaneous treatment with both lactate and α-CHCA did not significantly increase the CFP/YFP fluorescence ratio (**Figure 4G-H**). This phenomenon indicates that lactate is primarily transported into ECs through MCT1. Furthermore, lactate intervention at various concentrations of lactate in bEnd.3 cells. The results demonstrated that 20 and 40 mM lactate significantly promoted EC tube formation and migration in bEnd.3 cells; however, these processes were inhibited by high concentrations of lactate (**Figure S3C-F**). This phenomenon may be related to the lactate transport capacity of ECs. The lactate concentration of cells can be regulated through lactate transport, but excessive lactate levels may exceed the transport capacity of ECs, thereby leading to impaired endothelial function. These results indicate that lactate transport is a crucial pathway in regulating and balancing lactate concentrations within ECs. MCT1 is a key protein that facilitates lactate transport into ECs and its utilization in energy metabolism, enhances tube formation and migration capabilities, and promotes glycolysis. Therefore, an impaired lactate transport pathway has a significant impact on EC function and metabolism.

### Neurons utilize lactate to increase glycolysis and promote axonal regeneration

**Figure 5:**
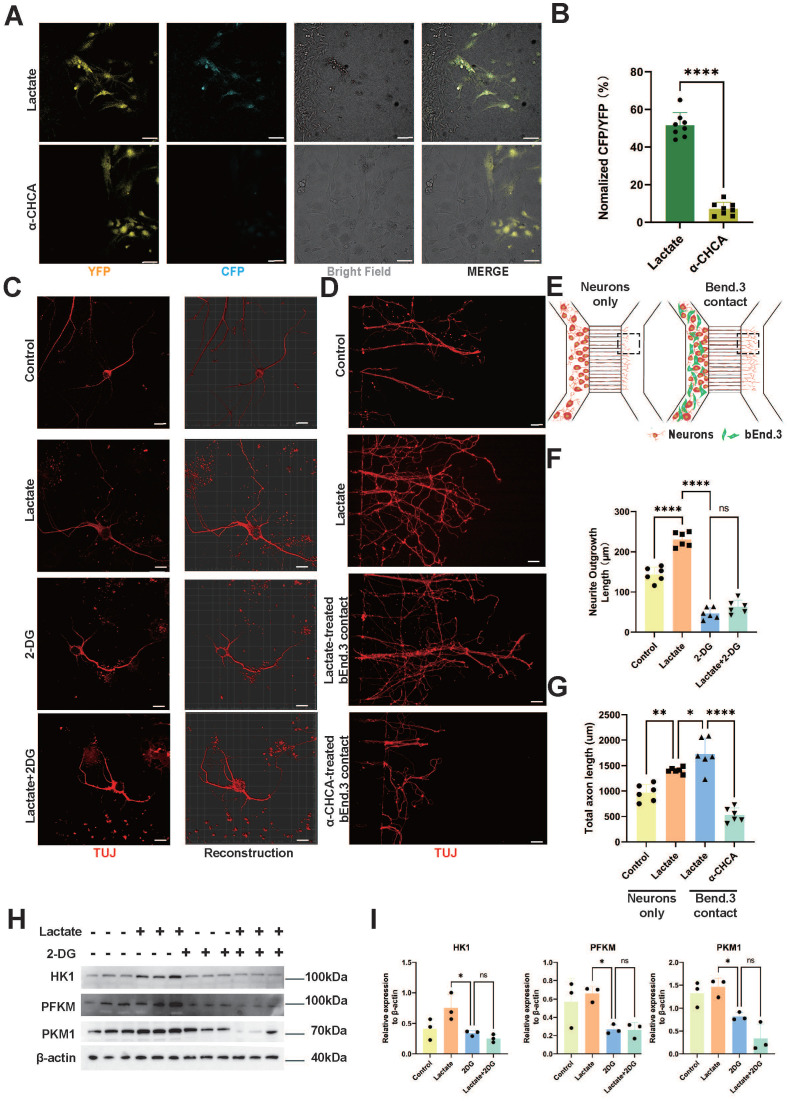
**Neurons Utilize Lactate to Enhance Glycolysis and Promote Axon Regeneration. (A)** Representative confocal image showing CFP (blue) and YFP (yellow), Bright field (white) fluorescence under lactate or α-CHCA treated bEnd.3 and laconic-infected cortical neurons on a 2-well chamber (scale bar = 100 μm). **(B)** Quantification of the CFP/YFP fluorescence ratio in (A) (n = 8 from 3 independent experiments, mean ± SD, unpaired t-test, ****p < 0.0001).** (C)** Representative immunofluorescent images of the regenerative axons under lactate or 2-DG treatment in four groups (red: TUJ11, scale bar = 20 μm). **(D)** Representative immunofluorescent images of the regenerative axons on the microfluidic culture plate under lactate or 2-DG treatment in four groups (red: Tuj1, scale bar = 100 μm). **(E)** Schematic illustration of the axon regeneration assay using the silicon-based microfluidics SND450. neuronal cytoplasm cultured alone and neuronal cytoplasm in contact with bEnd.3 are cultured in the left chamber, with axons growing along the channels to the observation area in the right chamber. The boxed area indicates the region for immunofluorescence imaging. **(F)** Quantification of total axon length in (C) (n = 6 from 3 independent experiments, mean ± SD, one-way ANOVA, ****p < 0.0001). **(G)** Quantification of total axonal length in (D) (n = 6 from 3 independent experiments, mean ± SD, one-way ANOVA, *p < 0.05, **p < 0.01, ****p < 0.0001). **(H)** Western blotting analysis of the levels of HK1, PFKM, PKM1 and β-actin in CATH.a Cells with different treatments. **(I)** Quantification of the relative expression of HK1, PFKM, PKM1 to β-actin in (G) (n = 3 from 3 independent experiments, mean ± SD, one-way ANOVA, ns not significant, *p < 0.05).

To determine whether neurons enhance glycolysis through lactate delivery by ECs, we cocultured bEnd.3 cells and Laconic lentivirus-infected cortical neurons in Ibidi chambers. Lactate or α-CHCA was added to the bEnd.3 side chamber. The inserts were removed 24 h postintervention to allow cell migration in the replaced regular culture medium without lactate or α-CHCA. Strikingly, the cortical neurons in contact with bEnd.3 cells presented an increased CFP/YFP ratio upon lactate treatment, as demonstrated by laser confocal microscopy (**Figure 5A-B**). Conversely, α-CHCA treatment decreased the CFP/YFP ratio, indicating a decrease in lactate entry from bEnd.3 cells into cortical neurons. Similar results were obtained by coculturing bEnd.3 cells with Laconic lentivirus-infected CATH.a cells (**Figure S4B, E**). An *in vitro* neurite outgrowth assay revealed that, compared with the control condition, lactate treatment elongated the neurites in cortical neurons, whereas the glycolysis inhibitor 2-DG treatment inhibited neurite growth. However, lactate treatment mitigated the inhibitory effect of 2-DG in combination with the other treatments on neurite growth (**Figure 5C, F**). Furthermore, CATH.a cells yielded similar results to those of the above assays (**Figure S4C, F**). To elucidate the lactate shuttle interaction between ECs and neurons, we used SND450 neural culture plates to isolate the neuronal cytoplasm and axons, and the distal axons were severed to simulate axonal rupture after SCI (**Figure 5D-E**). Subsequently, bEnd.3 cells treated with lactate or α-CHCA were cocultured with the neuronal cytoplasm. An axonal regenerative assay indicated that lactate treatment promoted the regeneration of cortical neurons. Additionally, treatment of the cortical neuronal cytoplasm with lactate alone promoted axon regeneration. The axon regeneration capacity was further enhanced when lactate-treated bEnd.3 cells were cocultured with the neuronal cytoplasm. However, regeneration was inhibited by α-CHCA. (**Figures 5D, G**). Additionally, the Western blot results demonstrated increased glycolytic ability in CATH.a after lactate treatment, whereas 2-DG treatment suppressed this effect (**Figure 5H-I**). Collectively, these findings indicate that neurons utilize lactate to increase their glycolytic capacity, thereby promoting axon regeneration. The MCT1-mediated lactate shuttle in ECs can enhance axon regeneration in neurons through direct contact.

### Overexpression of MCT1 in spinal cord ECs rescues impaired neurogenesis in SCI mice

**Figure 6:**
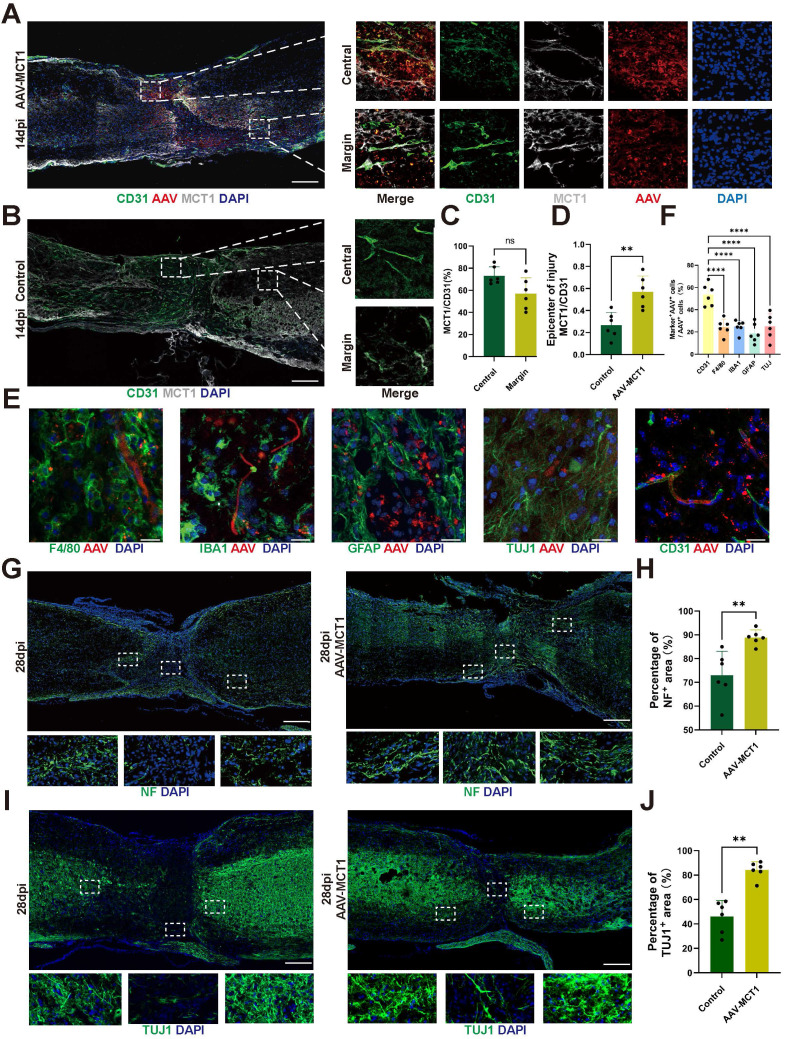
** Spinal Cord ECs Overexpression of MCT1 Rescues Impaired Neurogenesis in SCI Mice. (A)** Representative fluorescence images showing the colocalization of CD31, MCT1 with MCT1-AAV in the injured spinal cord at 14dpi, in the AAV-MCT1 treated group. And Localized enlargements of immunofluorescence staining. The upper panel shows the margin region of the injury site, and the lower panel shows the central region of the injury site (white: MCT1, red: AAV, green: CD31, blue: DAPI, scale bar: 200 μm). **(B)** Representative fluorescence images showing the colocalization of CD31, MCT1 with MCT1-AAV in the injured spinal cord at 14dpi, in the control group. The upper panel shows the margin region of the injury site, and the lower panel shows the central region of the injury site (white: MCT1, green: CD31, blue: DAPI, scale bar: 200 μm). **(C)** Quantification of the Colocalization of MCT1^+^ cells with CD31^+^ cells in the central and margin regions in (A) (n = 6 from 6 independent experiments, mean ± SD, unpaired t-test, ns not significant). **(D)** Quantification of the MCT1 area/ CD31 area in the central region of the injury site from (A, B) (n = 6 from 6 independent experiments, mean ± SD, unpaired t-test, **p < 0.01). **(E)** Representative immunofluorescence images of GFAP, TUJ1, F4/80, CD31 (green), and AAV (red) in the injury site (scale bar: 50 μm). **(F)** Quantification of the percentage of AAV^+^ cells in F4/80^+^, IBA1^+^, GFAP^+^, TUJ1^+^, and CD31^+^ cells in (E) (n = 6 from 6 independent experiments, mean ± SD, one-way ANOVA, ****p < 0.0001). **(G)** Representative immunofluorescent stains of NF images of the spinal cord at 28 dpi in each group (green: NF, scale bar: 200μm). **(H)** Quantification of NF positive signals in different areas roster to the epicenter of control, and AAV-MCT1 groups in (G) (n = 6 from 6 independent experiments, mean ± SD, unpaired t-test, **p < 0. 01).** (I)** Representative immunofluorescent stains of TUJ1 images of the spinal cord at 28 dpi in each group (scale bar: 200μm).** (J)** Quantification of TUJ1 positive signals in different areas roster to the epicenter of control, and AAV-MCT1 groups in (I) (n = 6 from 6 independent experiments, mean ± SD, unpaired t-test, **p < 0. 01).

To verify the relationship between the decrease in MCT1 in ECs and axon regeneration after SCI, we injected AAV-MCT1 under the control of the spinal cord EC-specific promoter TIE1p into the mouse spinal cord *in situ* to restore the expression of MCT1 in ECs at the epicenter of injury (**Figure 6A-D**). Immunofluorescence experiments confirmed that the AAV-MCT1 virus specifically expresses in the vascular ECs of mice in the Sham group. We also performed immunofluorescence staining for macrophages, microglia, astrocytes, and neurons in the spinal cord of mice injected with AAV-MCT1. The results showed that these cells did not co-localize with AAV-MCT1. (**Figure 6E-F**). Next, we assessed whether restoring MCT1 improves axonal regeneration in spinal cord ECs in SCI model mice. Neuronal axons were fluorescence-labeled with anti-neurofilament (NF) and anti-β-Tubulin III (TUJ1). Compared with the control, AAV-MCT1 injection promoted axon regeneration at the edge of the lesion core and increased the number of NF-, and TUJ1-positive signals in the spinal cord (**Figure 6G-J**). These results provide direct *in vivo* evidence that restored MCT1 expression in spinal cord ECs regulates lactate shuttling and axon regeneration in SCI mice.

### Spinal cord vasculature overexpression of MCT1 rescues impaired functional recovery in SCI mice

**Figure 7:**
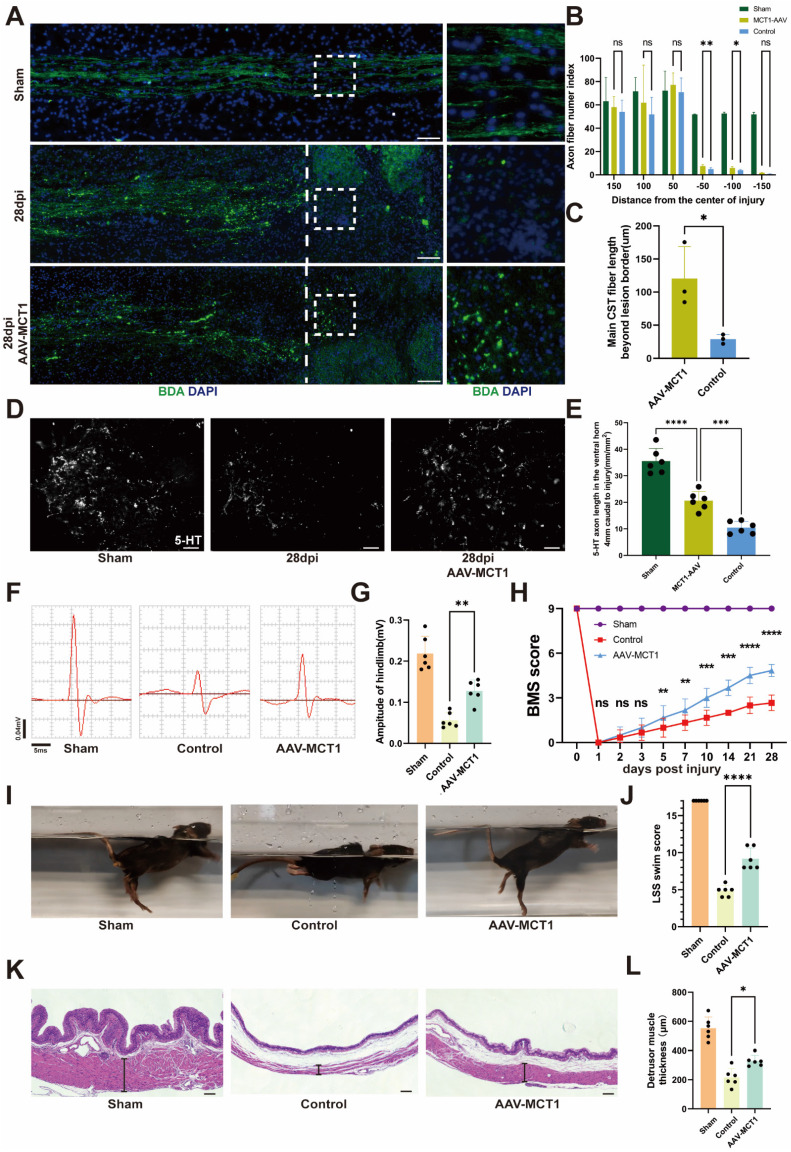
** MCT1 treated enhanced tissue repair and functional recovery after spinal cord injury. (A)** BDA labeling of the CST main tract in the dorsal column after SCI WT and AAV-MCT1 mice, 28 dpi. The dotted line indicates the lesion sites. **(B)** Quantification of CST axon (fiber) number index at various distances rostral to the lesion sites in (A) (n = 3 from 3 independent experiments, mean ± SD, two-way ANOVA, ns not significant, ∗∗ p < 0.01, *p < 0. 05).** (C)** Quantification of main CST fiber length beyond lesion border in (A) (n = 3 from 3 independent experiments, mean ± SD, unpaired t-test, *p < 0. 05).** (D)** Coronal view of 5-HT-immunoreactive raphespinal fibers in the spinal cord ventral horn of mice 4 mm caudal to the lesion 28dpi (scale bar=20 μm). **(E)** Quantification of 5-HT axon length in the ventral horn 4mm caudal to injury in (D) (n = 3 from 3 independent experiments, mean ± SD, one-way ANOVA, ***p < 0.001, **** p < 0.0001).** (F)** Representative images of hindlimb motor evoked potentials (MEPs) in the sham, control, and treatment groups at 28 dpi. **(G)** Quantification of the amplitude of hindlimb in (F) (n=6, mean ± SD, one-way ANOVA, ∗∗ p < 0.01). **(H)** Basso Mouse Scale (BMS) scores over time post-injury in the sham, control, and treatment groups (n = 6 from 6 independent experiments, mean ± SD, two-way ANOVA, Tukey's multiple comparisons, ns not significant, *p < 0.05, **p < 0.01, ***p < 0.001, **** p < 0.0001). **(I)** Representative images of swimming test at 28 dpi in the sham, control, and treatment groups. **(J)** Quantification of the swimming test in (I) using the Louisville Swim Scale (LSS) swim score (n=6, mean ± SD, one-way ANOVA, ***p < 0.01).** (K)** Representative images of hematoxylin and eosin staining (H&E) of the bladder (scale bar=200 μm) and bladder macroscopic photo at 28 dpi in the sham, control, and treatment groups. **(L)** Quantification of the detrusor muscle thickness in (K) (n = 6 from 6 independent experiments, mean ± SD, one-way ANOVA, Tukey's multiple comparisons, *p < 0.05).

Following AAV-MCT1 injection, we used an anterograde tracer, biotinylated dextran amine (BDA), which was injected into the sensorimotor cortex to trace the descending CST axons 28 dpi. In contrast, many BDA-labeled CST axons regenerated robustly through the lesion and extended to the caudal spinal cord in AAV-MCT1-treated SCI mice (**Figure 7A-C**). We then quantified the density of RST axons at 28 dpi in the AAV-MCT1-treated SCI mice used for CST analysis by immunohistochemistry to assess 5-hydroxytryptamine (5-HT) labeling. The results showed a significantly higher density of RST axons compared to the control group (**Figure 7D-E**). Electrophysiological tests revealed a significant increase in the motor-evoked potential (MEP) amplitude in the hind limb of SCI mice 28 dpi (**Figure 7F-G**). Hind limb motor function was assessed via the Basso Mouse Scale (BMS), and the scores indicated an improved recovery in AAV-MCT1-treated SCI mice starting at 7 dpi. By 28 dpi, the hind limb motor function of treated SCI mice had markedly improved (**Figure 7H**). Swimming tests demonstrated more stable trunk movements, fewer tilted body angles, and fewer drooping tails in the AAV-MCT1 group, resulting in a significantly elevated LSS swimming score (**Figure 7I-J**). Moreover, analysis of hematoxylin-eosin-stained slices from SCI mouse bladders revealed a significant increase in the thickness of the detrusor muscle layer in the neurogenic bladder following AAV-MCT1 treatment, suggesting the restoration of nerve innervation in the bladder (**Figure 7K-L**). Overall, our findings confirmed the positive therapeutic effect of restoring MCT1 expression in spinal cord ECs on functional recovery in SCI mice.

## Discussion

The results of this study demonstrate that endothelium-derived lactate can be uptaken and utilized by neurons for energy generation and axonal regeneration, which is crucial for maintaining normal neuron function and axon regeneration. After SCI, there is abnormal lactate metabolism at the injury epicenter, where EC destruction leads to obstruction of the lactate shuttle. Additionally, we identified MCT1 as the EC-specific lactate transporter. By upregulating the expression of MCT1 in ECs, we restored lactate transport function, enhanced neuronal glycolysis, promoting axon regeneration and neurological function recovery after SCI. It highlights that MCT1 serves as a novel therapeutic target for SCI, providing a new direction after SCI treatment from a metabolic perspective.

Metabolic dysfunction is a key pathophysiological process in secondary injury following SCI 2. Sustained microvascular thrombosis and spasms at the injury epicenter cause further neuron death. In our previously published work from our team, we have demonstrated persistent impairment of barrier function in blood vessels within the injury site 35. Together with the findings of MCT1 expression loss in this study, these aspects collectively illustrate the pathological changes in vasculature following SCI. This work, from a metabolic perspective, investigates whether the loss of microvascular function after SCI leads to metabolic changes in the epicenter of injury. Energy metabolomic analysis of mice after SCI reveals an elevation in glycolysis-related metabolites, with lactate being a significantly increased metabolite post-injury. Existing literature suggests that ECs can maintain central nervous system energy metabolism by transporting lactate, thereby regulating neuronal functional stability 36. Meanwhile, enhancing glycolysis in glial cells can promote axon regeneration at the epicenter of injury 14, and there is also literature suggesting that increasing the neuronal intrinsic glycolytic capacity can inhibit neuronal apoptosis 37. Therefore, focusing on metabolic changes at the site of injury after SCI may provide new research directions for studying neurological function recovery post-SCI.

Restoring cellular energy metabolism after SCI can promote axon regeneration and functional recovery 24, 38. Lactate has been widely accepted as a metabolic waste product; however, recent studies have shown that lactate is a crucial fuel in energy metabolism 39. In the central nervous system, lactate serves as a vital source of energy, although its contribution to the tricarboxylic acid (TCA) cycle compared to glucose remains a contentious issue 40. Neurons can uptake extracellular lactate as an important supplement for their energy metabolism, fulfilling the increased demand for energy during synaptic activity enhancement.

Under conditions of sufficient oxygen, lactate can be converted back to pyruvate via the reverse action of lactate dehydrogenase, entering aerobic metabolic pathways to provide energy as a nutrient for cells. On the other hand, lactate may serve as a signaling molecule that induces the expression of immediate early genes through activation of specific receptors and downstream signaling pathways 41, 42. The roles of lactate in the delivery of oxidative and gluconeogenic substrates as well as in cell signaling is termed the lactate shuttle 43. Research on lactate shuttle initially focused on describing and analyzing the whole organ lactate shuttle mechanisms 44, 45. However, cellular lactate shuttle pathways have garnered increasing attention in recent years. Two recent studies have indicated that pericytes and macrophages can fulfill their energy requirements by receiving lactate from ECs. Endothelial-derived lactate is absorbed by pericytes to maintain blood-brain barrier stability 46, while lactate produced by ECs induces macrophage polarization to promote muscle regeneration following ischemic injury 47. Restoring cellular glycolysis is a crucial pathway for promoting axonal regeneration and functional recovery after SCI 48.

Furthermore, ECs can also utilize lactate as a driving force, along with high rates of glycolysis, to promote angiogenesis 49. We found that lactate concentrations below 40 mM promote bEnd.3 endothelial cell functions, whereas 100 mM lactate exhibited cytotoxicity, impairing tube formation and migration. High lactate concentrations may also induce endothelial cell phenotypic transition, hindering tissue repair 50. Additionally, we discovered that neurons rely on endothelium-shuttled lactate to provide energy. This is crucial for studying lactate shuttle and functional recovery of neurovascular after SCI, as a lack of endothelium-derived lactate can lead to impediments in axon regeneration and diminished neurological recovery. These findings provide a basis for therapeutic strategies aiming to shuttle lactate from endothelial cells to neurons: 1) facilitating axonal regeneration and 2) maintaining endothelial lactate homeostasis.

Endothelium-derived lactate is present at high concentrations locally, it becomes an easily accessible fuel source around blood vessels 51. Neurons and ECs are typically in direct contact throughout the entire neurovascular network, endothelium-derived lactate may be the main source of energy supply for neurons. Lactate-related transport proteins are indispensable for mediating the lactate shuttle between cells 52. The specific MCT members responsible for lactate uptake in neurons, their efficiency in lactate uptake, and their specific functions *in vivo* still require further investigation. In the rat hippocampus, neurons can uptake lactate through MCT2 18. As for how spinal cord neurons uptake lactate, we currently lack an answer and further research is needed in the future. However, our data suggest that MCT1, a lactate transporter protein, is specifically expressed in ECs within the spinal cord, playing a crucial role in mediating the transport of lactate across endothelial cells. Regarding how lactate is transported from ECs into neurons, we found that this transport occurs exclusively through direct contact between the two. In the presence of direct contact with endothelial cells, lactate can enter neurons more efficiently, leading to a sustained increase in cellular lactate.

After SCI, insufficient axon regeneration often leads to poor recovery, representing one of the most pressing challenges in SCI treatment. Developing successful regenerative strategies to reconnect axons within the central nervous system is paramount for SCI research. It is widely believed that inadequate intrinsic neuronal growth capacity, a deficient growth environment, and the absence of neurotrophic factors are the main reasons for hindered axonal regeneration 53, 54. Currently, while there is considerable research on axonal regeneration in adults 55-57, the translation of these findings into clinically applicable therapeutic methods is severely limited. Metabolic reprogramming emerges as a novel strategy to stimulate axonal regeneration in the central nervous system 58, 59. Previous studies have shown that lactate serves as an energy source for neurons 60, and local application of lactate to the injured spinal cord can promote CST axonal regeneration 14. Our research further reveals an unexpected connection between EC-neuron lactate shuttling after SCI and neural functional recovery, offering a novel metabolic target for SCI treatment. We found that impaired lactate shuttling due to the lack of MCT1 in ECs at the epicenter of injury impedes neuronal energy metabolism, resulting in weakened axonal regeneration capacity. Restoring MCT1 expression in ECs at the epicenter of injury significantly promotes axon regeneration and functional recovery. Additionally, we found that newly formed blood vessels after SCI not only exhibit a loss of key proteins such as MCT1, but their morphology also differs from that of normal spinal cord vasculature, often appearing as large, abnormal vessels. Previous research by our team has also found that repairing and stabilizing the BSCB after SCI aids in the recovery of neural function 61. Therefore, whether these vascular morphological changes and the loss of key proteins adversely affect recovery after SCI, how the newly formed blood vessels differ from normal ones, and whether restoring normal vascular morphology can further aid in the recovery of the BSCB, remain subjects for further investigation. While we did not specifically study whether other cells transport lactate to neurons after SCI, besides ECs, other cells may also contribute. For instance, it has been reported that astrocytes secrete lactate to fuel neuronal mitochondria in traumatic brain injury 62. Interestingly, lactate not only serves as a metabolic substrate but also functions as a signaling molecule to modulate neuronal function 60, 63, 64. Whether this also promotes axonal regeneration warrants further investigation.

## 5. Conclusions

In summary, our study demonstrates that lactate produced by ECs is utilized by adjacent neurons for energy metabolism and axonal regeneration. The impaired lactate metabolism observed in mice following SCI is attributed to the lack of MCT1 in ECs at the epicenter of injury. Restoring MCT1 expression at the lesion site in adult mammals promotes significant axonal regeneration and functional recovery.

## Supplementary Material

Supplementary figures.

Supplementary movie 1.

Supplementary movie 2.

## Abbreviations

AAV: Adeno-associated virus
α-CHCA: alpha-cyano-4-hydroxycinnamic acid
ANLS: astrocyte-neuron lactate shuttle
BDA: biotin-dextran amine
BMS: Basso Mouse Scale
BSCB: blood-spinal cord barrier
CFP/YFP: yellow fluorescence protein/cyan fluorescence protein
CST: corticospinal tract
dpi: days post-injury
ECs: Endothelial cells
HK1: hexokinase 1
KEGG: Kyoto Encyclopedia of Genes and Genomes
LSS: Louisville Swim Scale
MCTs: Monocarboxylic transporters
MCT1: monocarboxylate transporter 1
MEP: motor-evoked potential
NF: neurofilament
PFKM: phosphofructokinase 1
PKM1: pyruvate kinase 1
SCI: Spinal cord injury
SD: standard deviation
TUJ1: β-Tubulin III
2-DG: 2-Deoxy-D-glucose

## References

[1] Global regional, national burden of spinal cord injury 1990-2019 (2023). a systematic analysis for the Global Burden of Disease Study 2019. Lancet Neurol.

[2] Ahuja CS, Wilson JR, Nori S, Kotter MRN, Druschel C, Curt A (2017). Traumatic spinal cord injury. Nat Rev Dis Primers.

[3] Mautes AE, Weinzierl MR, Donovan F, Noble LJ (2000). Vascular events after spinal cord injury: contribution to secondary pathogenesis. Phys Ther.

[4] LaPlaca MC, Simon CM, Prado GR, Cullen DK (2007). CNS injury biomechanics and experimental models. Prog Brain Res.

[5] Tomko P, Farkaš D, Čížková D, Vanický I (2017). Longitudinal enlargement of the lesion after spinal cord injury in the rat: a consequence of malignant edema?. Spinal Cord.

[6] Lemke M, Demediuk P, McIntosh TK, Vink R, Faden AI (1987). Alterations in tissue Mg++, Na+ and spinal cord edema following impact trauma in rats. Biochem Biophys Res Commun.

[7] Yamada S, Sanders DC, Maeda G (1981). Oxidative metabolism during and following ischemia of cat spinal cord. Neurol Res.

[8] Slater PG, Domínguez-Romero ME, Villarreal M, Eisner V, Larraín J (2022). Mitochondrial function in spinal cord injury and regeneration. Cell Mol Life Sci.

[9] Cheng L, Cai B, Lu D, Zeng H (2023). The role of mitochondrial energy metabolism in neuroprotection and axonal regeneration after spinal cord injury. Mitochondrion.

[10] He Z, Du J, Zhang Y, Xu Y, Huang Q, Zhou Q (2023). Kruppel-like factor 2 contributes to blood-spinal cord barrier integrity and functional recovery from spinal cord injury by augmenting autophagic flux. Theranostics.

[11] Jin LY, Li J, Wang KF, Xia WW, Zhu ZQ, Wang CR (2021). Blood-Spinal Cord Barrier in Spinal Cord Injury: A Review. J Neurotrauma.

[12] Zhou R, Li J, Chen Z, Wang R, Shen Y, Zhang R (2023). Pathological hemodynamic changes and leukocyte transmigration disrupt the blood-spinal cord barrier after spinal cord injury. J Neuroinflammation.

[13] Kang CE, Clarkson R, Tator CH, Yeung IW, Shoichet MS (2010). Spinal cord blood flow and blood vessel permeability measured by dynamic computed tomography imaging in rats after localized delivery of fibroblast growth factor. J Neurotrauma.

[14] Li F, Sami A, Noristani HN, Slattery K, Qiu J, Groves T (2020). Glial Metabolic Rewiring Promotes Axon Regeneration and Functional Recovery in the Central Nervous System. Cell Metab.

[15] Wei Y, Miao Q, Zhang Q, Mao S, Li M, Xu X (2023). Aerobic glycolysis is the predominant means of glucose metabolism in neuronal somata, which protects against oxidative damage. Nat Neurosci.

[16] Yang C, Pan RY, Guan F, Yuan Z (2024). Lactate metabolism in neurodegenerative diseases. Neural Regen Res.

[17] Veloz Castillo MF, Magistretti PJ, Calì C (2021). l-Lactate: Food for Thoughts, Memory and Behavior. Metabolites.

[18] Suzuki A, Stern SA, Bozdagi O, Huntley GW, Walker RH, Magistretti PJ (2011). Astrocyte-neuron lactate transport is required for long-term memory formation. Cell.

[19] Miyamoto K, Ishikura KI, Kume K, Ohsawa M (2019). Astrocyte-neuron lactate shuttle sensitizes nociceptive transmission in the spinal cord. Glia.

[20] Ardanaz CG, Ramírez MJ, Solas M (2022). Brain Metabolic Alterations in Alzheimer's Disease. Int J Mol Sci.

[21] Hashimoto T, Hussien R, Cho HS, Kaufer D, Brooks GA (2008). Evidence for the mitochondrial lactate oxidation complex in rat neurons: demonstration of an essential component of brain lactate shuttles. PLoS One.

[22] Takeuchi H, Suzuki M, Goto R, Tezuka K, Fuchs H, Ishiguro N (2022). Regional Differences in the Absolute Abundance of Transporters, Receptors and Tight Junction Molecules at the Blood-Arachnoid Barrier and Blood-Spinal Cord Barrier among Cervical, Thoracic and Lumbar Spines in Dogs. Pharm Res.

[23] Park JW, Vahidi B, Taylor AM, Rhee SW, Jeon NL (2006). Microfluidic culture platform for neuroscience research. Nat Protoc.

[24] Han Q, Xie Y, Ordaz JD, Huh AJ, Huang N, Wu W (2020). Restoring Cellular Energetics Promotes Axonal Regeneration and Functional Recovery after Spinal Cord Injury. Cell Metab.

[25] Milich LM, Choi JS, Ryan C, Cerqueira SR, Benavides S, Yahn SL (2021). Single-cell analysis of the cellular heterogeneity and interactions in the injured mouse spinal cord. J Exp Med.

[26] Satija R, Farrell JA, Gennert D, Schier AF, Regev A (2015). Spatial reconstruction of single-cell gene expression data. Nat Biotechnol.

[27] Yadav A, Matson KJE, Li L, Hua I, Petrescu J, Kang K (2023). A cellular taxonomy of the adult human spinal cord. Neuron.

[28] Smith RR, Burke DA, Baldini AD, Shum-Siu A, Baltzley R, Bunger M (2006). The Louisville Swim Scale: a novel assessment of hindlimb function following spinal cord injury in adult rats. J Neurotrauma.

[29] Pandit R, Chen L, Götz J (2020). The blood-brain barrier: Physiology and strategies for drug delivery. Adv Drug Deliv Rev.

[30] San Martín A, Ceballo S, Ruminot I, Lerchundi R, Frommer WB, Barros LF (2013). A genetically encoded FRET lactate sensor and its use to detect the Warburg effect in single cancer cells. PLoS One.

[31] Halestrap AP, Meredith D (2004). The SLC16 gene family-from monocarboxylate transporters (MCTs) to aromatic amino acid transporters and beyond. Pflugers Arch.

[32] Pellerin L, Pellegri G, Martin JL, Magistretti PJ (1998). Expression of monocarboxylate transporter mRNAs in mouse brain: support for a distinct role of lactate as an energy substrate for the neonatal vs. adult brain. Proc Natl Acad Sci U S A.

[33] Kim D, Ko HY, Chung JI, Park YM, Lee S, Kim SY (2024). Visualizing cancer-originating acetate uptake through monocarboxylate transporter 1 in reactive astrocytes in the glioblastoma tumor microenvironment. Neuro Oncol.

[34] Nam MH, Ko HY, Kim D, Lee S, Park YM, Hyeon SJ (2023). Visualizing reactive astrocyte-neuron interaction in Alzheimer's disease using 11C-acetate and 18F-FDG. Brain.

[35] Xie Y, Luo Z, Peng W, Liu Y, Yuan F, Xu J (2023). Inhibition of UTX/KDM6A improves recovery of spinal cord injury by attenuating BSCB permeability and macrophage infiltration through the MLCK/p-MLC pathway. J Neuroinflammation.

[36] Wang J, Cui Y, Yu Z, Wang W, Cheng X, Ji W (2019). Brain Endothelial Cells Maintain Lactate Homeostasis and Control Adult Hippocampal Neurogenesis. Cell Stem Cell.

[37] Gao L, Wang C, Qin B, Li T, Xu W, Lenahan C (2020). 6-phosphofructo-2-kinase/fructose-2,6-bisphosphatase Suppresses Neuronal Apoptosis by Increasing Glycolysis and "cyclin-dependent kinase 1-Mediated Phosphorylation of p27 After Traumatic Spinal Cord Injury in Rats. Cell Transplant.

[38] Xu J, Shi C, Yuan F, Ding Y, Xie Y, Liu Y (2024). Targeted transplantation of engineered mitochondrial compound promotes functional recovery after spinal cord injury by enhancing macrophage phagocytosis. Bioact Mater.

[39] Rabinowitz JD, Enerbäck S (2020). Lactate: the ugly duckling of energy metabolism. Nat Metab.

[40] Hui S, Cowan AJ, Zeng X, Yang L, TeSlaa T, Li X (2020). Quantitative Fluxomics of Circulating Metabolites. Cell Metab.

[41] Xue X, Liu B, Hu J, Bian X, Lou S (2022). The potential mechanisms of lactate in mediating exercise-enhanced cognitive function: a dual role as an energy supply substrate and a signaling molecule. Nutr Metab (Lond).

[42] Jia L, Liao M, Mou A, Zheng Q, Yang W, Yu Z (2021). Rheb-regulated mitochondrial pyruvate metabolism of Schwann cells linked to axon stability. Dev Cell.

[43] Brooks GA (2018). The Science and Translation of Lactate Shuttle Theory. Cell Metab.

[44] Emhoff CA, Messonnier LA, Horning MA, Fattor JA, Carlson TJ, Brooks GA (2013). Gluconeogenesis and hepatic glycogenolysis during exercise at the lactate threshold. J Appl Physiol (1985).

[45] Glenn TC, Martin NA, Horning MA, McArthur DL, Hovda DA, Vespa P (2015). Lactate: brain fuel in human traumatic brain injury: a comparison with normal healthy control subjects. J Neurotrauma.

[46] Lee HW, Xu Y, Zhu X, Jang C, Choi W, Bae H (2022). Endothelium-derived lactate is required for pericyte function and blood-brain barrier maintenance. Embo j.

[47] Zhang J, Muri J, Fitzgerald G, Gorski T, Gianni-Barrera R, Masschelein E (2020). Endothelial Lactate Controls Muscle Regeneration from Ischemia by Inducing M2-like Macrophage Polarization. Cell Metab.

[48] De Bock K, Georgiadou M, Schoors S, Kuchnio A, Wong BW, Cantelmo AR (2013). Role of PFKFB3-driven glycolysis in vessel sprouting. Cell.

[49] Polet F, Feron O (2013). Endothelial cell metabolism and tumour angiogenesis: glucose and glutamine as essential fuels and lactate as the driving force. J Intern Med.

[50] Fan M, Yang K, Wang X, Chen L, Gill PS, Ha T (2023). Lactate promotes endothelial-to-mesenchymal transition via Snail1 lactylation after myocardial infarction. Sci Adv.

[51] Iadecola C, Beitz AJ, Renno W, Xu X, Mayer B, Zhang F (1993). Nitric oxide synthase-containing neural processes on large cerebral arteries and cerebral microvessels. Brain Res.

[52] Poole RC, Halestrap AP (1993). Transport of lactate and other monocarboxylates across mammalian plasma membranes. Am J Physiol.

[53] Lu Y, Belin S, He Z (2014). Signaling regulations of neuronal regenerative ability. Curr Opin Neurobiol.

[54] Anderson MA, O'Shea TM, Burda JE, Ao Y, Barlatey SL, Bernstein AM (2018). Required growth facilitators propel axon regeneration across complete spinal cord injury. Nature.

[55] Hutson TH, Kathe C, Palmisano I, Bartholdi K, Hervera A, De Virgiliis F (2019). Cbp-dependent histone acetylation mediates axon regeneration induced by environmental enrichment in rodent spinal cord injury models. Sci Transl Med.

[56] Kromer LF (1987). Nerve growth factor treatment after brain injury prevents neuronal death. Science.

[57] Dias DO, Kim H, Holl D, Werne Solnestam B, Lundeberg J, Carlén M (2018). Reducing Pericyte-Derived Scarring Promotes Recovery after Spinal Cord Injury. Cell.

[58] Huang N, Li S, Xie Y, Han Q, Xu XM, Sheng ZH (2021). Reprogramming an energetic AKT-PAK5 axis boosts axon energy supply and facilitates neuron survival and regeneration after injury and ischemia. Curr Biol.

[59] Huang N, Sheng ZH (2022). Microfluidic devices as model platforms of CNS injury-ischemia to study axonal regeneration by regulating mitochondrial transport and bioenergetic metabolism. Cell Regen.

[60] Magistretti PJ, Allaman I (2018). Lactate in the brain: from metabolic end-product to signalling molecule. Nat Rev Neurosci.

[61] Xie Y, Sun Y, Liu Y, Zhao J, Liu Q, Xu J (2023). Targeted Delivery of RGD-CD146(+)CD271(+) Human Umbilical Cord Mesenchymal Stem Cell-Derived Exosomes Promotes Blood-Spinal Cord Barrier Repair after Spinal Cord Injury. ACS Nano.

[62] Yokobori S, Mazzeo AT, Gajavelli S, Bullock MR (2014). Mitochondrial neuroprotection in traumatic brain injury: rationale and therapeutic strategies. CNS Neurol Disord Drug Targets.

[63] Yang J, Ruchti E, Petit JM, Jourdain P, Grenningloh G, Allaman I (2014). Lactate promotes plasticity gene expression by potentiating NMDA signaling in neurons. Proc Natl Acad Sci U S A.

[64] Tu NH, Katano T, Matsumura S, Funatsu N, Pham VM, Fujisawa JI (2017). Na(+) /K(+) -ATPase coupled to endothelin receptor type B stimulates peripheral nerve regeneration via lactate signalling. Eur J Neurosci.

