# Supplementary material for: MCT1-mediated endothelial cell lactate shuttle as a target for promoting axon regeneration after spinal cord injury
Source: Theranostics. 2024 Sep 3;14(14):5662–81. doi: 10.7150/thno.96374 (PMC11413787; doi:10.7150/thno.96374)
Supplement: Supplementary file 1 — Supplementary figures. [file thnov14p5662s1.pdf]

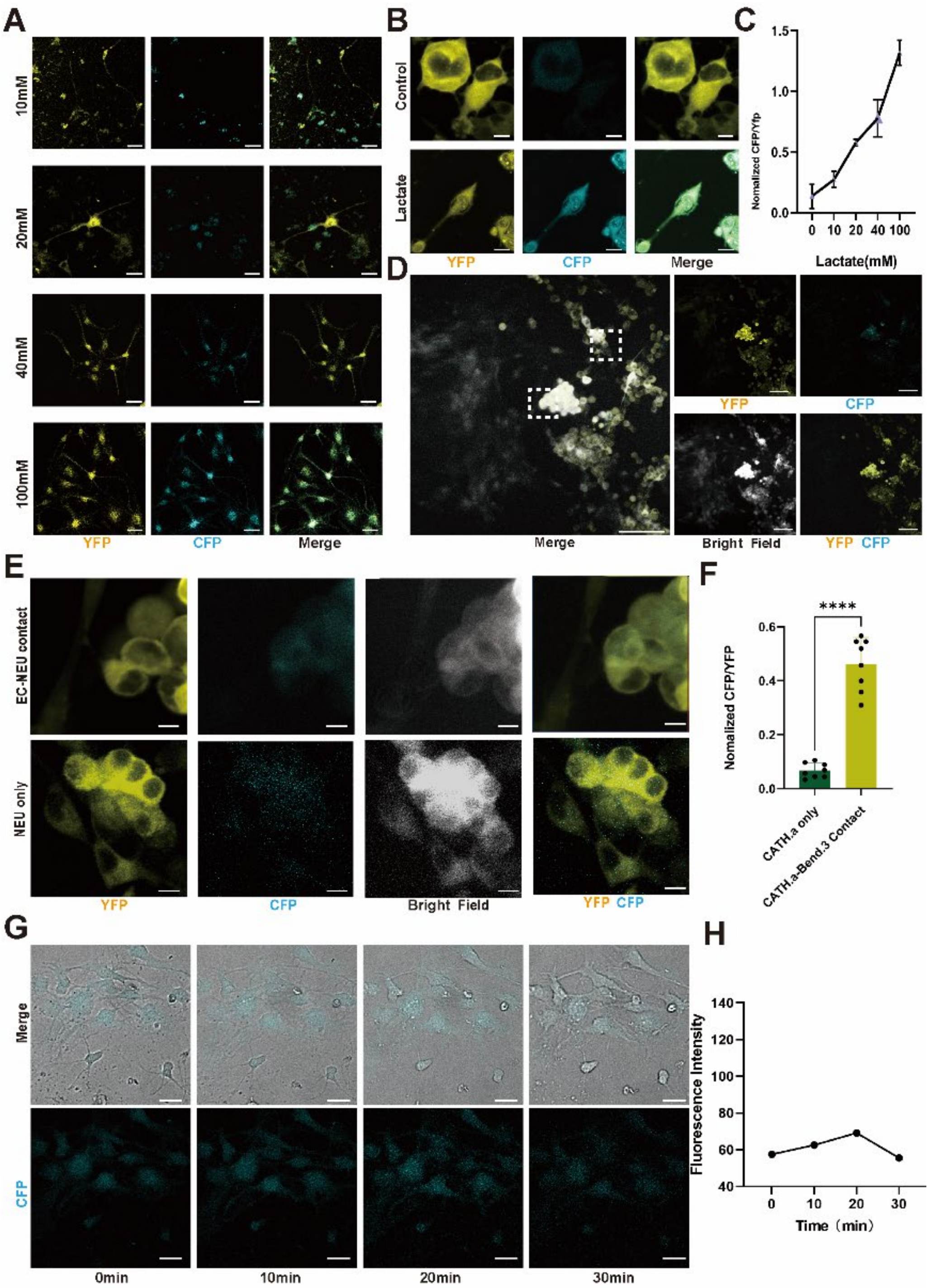

Supplementary Figure 1. Endothelium-derived lactate feeds nerve cells. (A) Immunofluorescence of Laconic lentivirus-infected cortical neurons treated with different concentrations of lactate. **(B)** Representative images showing CFP (blue) and YFP (yellow) fluorescence in control and lactate (100 mM) -treated CATH.a. (scale bar = 10μm). **(C)** Quantification of the CFP/YFP fluorescence ratio of laconic (lactate sensor) showing concentration-dependent lactate uptake in CATH.a. (n = 6 from 3 independent experiments. Error bars indicate the standard error of the mean (SEM). **(D)** Representative confocal image showing CFP (blue) and YFP (yellow), Bright field (white) fluorescence from bEnd.3 and laconic-infected CATH.a on a 2-well

chamber. (scale bar = 100 μm). **(E)** Localized enlargements showing CFP (blue) and YFP (yellow), Bright field (white) fluorescence from bEnd.3 and laconic-infected CATH.a on a 2-well chamber. The upper panel shows CATH.a with endothelial contact and the lower panel shows CATH.a without endothelial contact (scale bar = 10 μm). **(F)** Quantification of the CFP/YFP fluorescence ratio of laconic in CATH.a with endothelial contact (EC-NEU contact) or CATH.a alone (NEU only) (n = 8 from 3 independent experiments. Error bars indicate the standard error of the mean (SEM) from unpaired Student's t-test, **** $p < 0.0001$). **(G)** Representative time-lapse imaging at 0, 10, 20, and 30 min, along with CFP (blue), bright field (white) fluorescence from laconic-infected cortical neurons alone on a 2-well chamber. (scale bar = 20 μm). **(H)** Quantification of the CFP fluorescence ratio of laconic showing time-dependent lactate transport from bEnd.3 to cortical neurons.

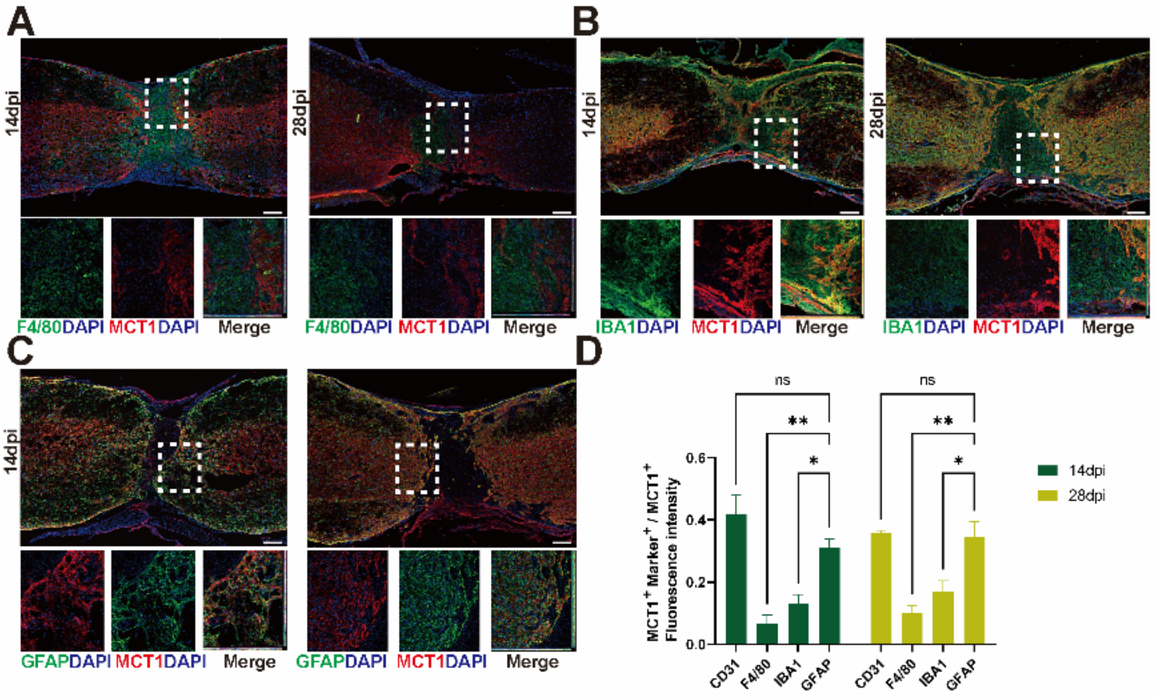

Supplementary Figure 2. Spatial and temporal expression of MCT1 after SCI. (A) Representative fluorescence images showing the expression of MCT1 in the spinal cord of the 14, 28 dpi groups (red: MCT1, green: F4/80, blue: DAPI, scale bar: 200 µm). **(B)** Representative fluorescence images showing the expression of MCT1 in the spinal cord of the 14, 28 dpi groups (red: MCT1, green: IBA1, blue: DAPI, scale bar: 200 µm). **(C)** Representative fluorescence images showing the expression of MCT1 in the spinal cord of the 14, 28 dpi groups (red: MCT1, green: GFAP, blue: DAPI, scale bar: 200 µm). **(D)** Quantification of the Colocalization of $MCT1^+$ $Marker^+$ cells with $MCT1^+$ cells in (A-C) of the 14, 28 dpi groups. (n = 8 from 6 independent experiments, mean ± SD, two-way ANOVA, Tukey's multiple comparisons, ns no significant, *p < 0.05, *p < 0.01).

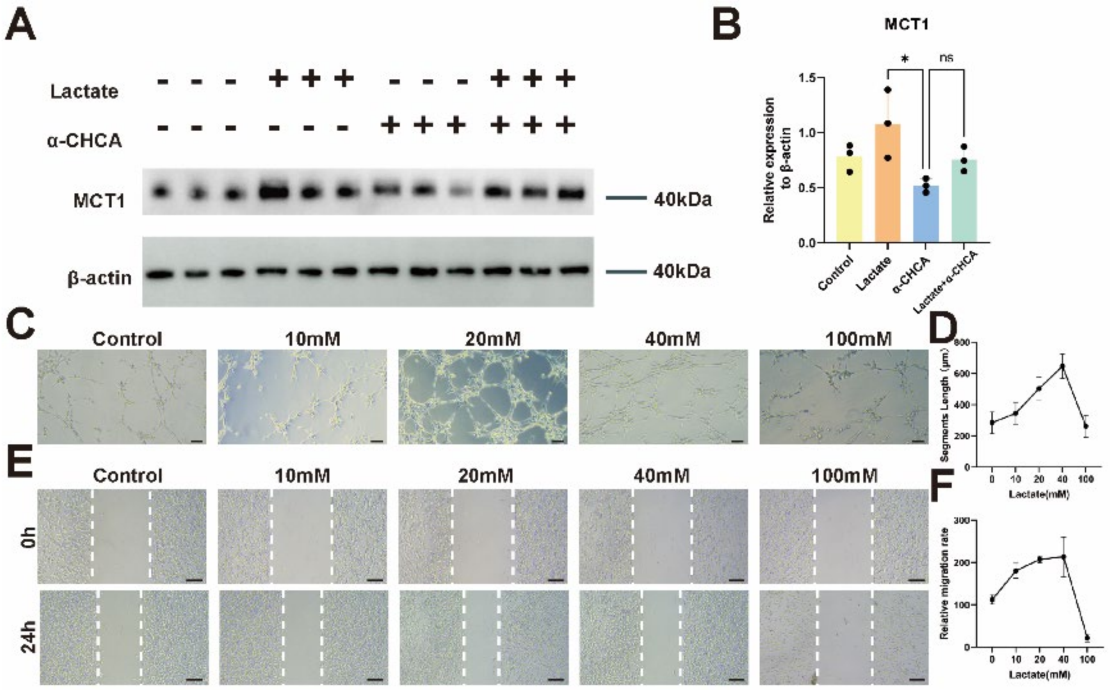

Supplementary Figure 3. Inhibition of ECs MCT1 Expression Leads to Decreased Biological and Metabolic Functions. (A) Western blotting analysis of the levels of MCT1, and β-actin in bEnd.3 Cells with different concentrations of lactate. **(B)** Quantification of the relative expression of MCT1 to β-actin in (A) (n = 3 from 3 independent experiments, mean ± SD, one-way ANOVA, ns not significant, *p < 0.05). **(C)** Representative images showing the tube-forming ability of bEnd.3 Cells with different concentrations of lactate (scale bar = 100 μm). **(D)** Quantification of tube-forming ability in (C). (n = 6 from 3 independent experiments, mean ± SD, one-way ANOVA). **(E)** Representative images showing the lateral migration ability of bEnd.3 Cells with different concentrations of lactate (scale bar = 100 μm). **(F)** Quantification of tube-forming ability in (E) (n = 3 from 3 independent experiments, mean ± SD, one-way ANOVA).

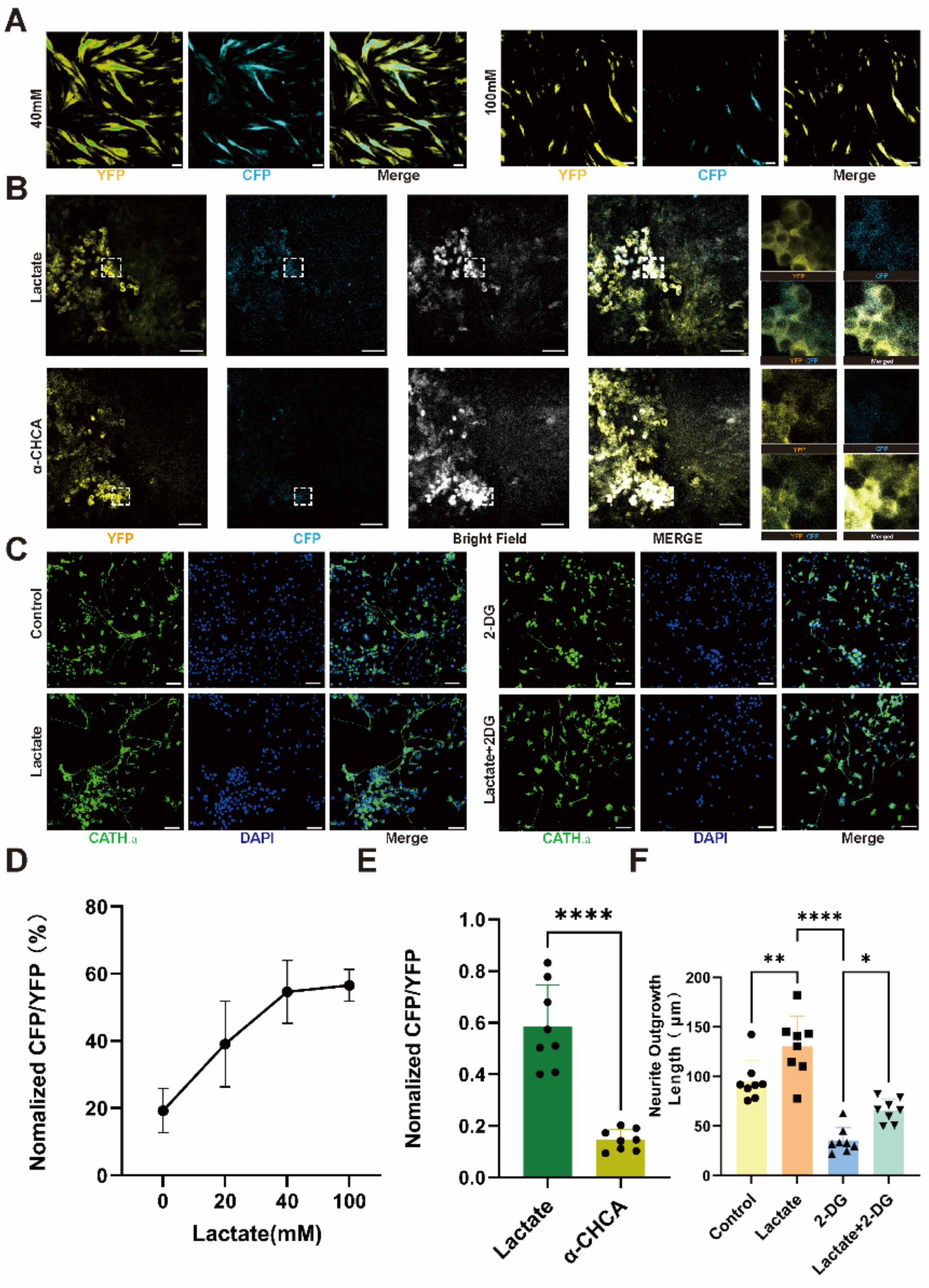

Supplementary Figure 4. (A) Immunofluorescence of Laconic lentivirus-infected bEnd.3 treated with different concentrations of lactate. **(B)** Representative confocal image showing CFP (blue) and YFP (yellow), Bright field (white) fluorescence from lactate or α-CHCA treated bEnd.3 and laconic-infected CATH.a on a 2-well chamber. (scale bar = 100 μm). **(C)** Representative immunofluorescent images of the regenerative axons under lactate or 2-DG treatment in four groups (green: Tuj1, blue:

DAPI, scale bar = 100 μm). **(D)** Quantification of the CFP/YFP fluorescence ratio of laconic (lactate sensor) showing concentration-dependent lactate uptake in bEnd.3. (n = 6 from 3 independent experiments. Error bars indicate the SEM). **(E)** Quantification of the CFP/YFP fluorescence ratio of laconic in CATH.a with bEnd.3 under lactate treatment or under α-CHCA treatment in (B) (n = 8 from 3 independent experiments, mean ± SD, unpaired t-test, ****$p < 0.0001$). **(F)** Quantification of total axon length in (C) (n = 8 from 3 independent experiments, mean ± SD, one-way ANOVA, *$p < 0.05$, **$p < 0.01$, ***$p < 0.001$).
